# Unexpected Inheritance Patterns in a Large Cohort of Patients with a Suspected Ciliopathy

**DOI:** 10.1155/2023/2564200

**Published:** 2023-08-09

**Authors:** Aurélie Gouronc, Elodie Javey, Anne-Sophie Leuvrey, Elsa Nourisson, Sylvie Friedmann, Valérie Reichert, Nicolas Derive, Christine Francannet, Boris Keren, Jonathan Lévy, Marc Planes, Lyse Ruaud, Jeanne Amiel, Hélène Dollfus, Sophie Scheidecker, Jean Muller

**Affiliations:** ^1^Genetics Diagnostic Laboratory, Strasbourg University Hospital, Strasbourg, France; ^2^Medical Biology Laboratory SeqOIA-PFMG2025, Paris, France; ^3^Medical Genetics Service, Clermont-Ferrand University Hospital, Clermont-Ferrand, France; ^4^Genetics Department, Pitié-Salpêtrière Hospital, AP-HP, Sorbonne University, Paris, France; ^5^Genetics Department, Robert Debré University Hospital, APHP Nord, Paris, France; ^6^Clinical Genetics Service, CHRU Morvan, 29609 Brest, France; ^7^UMR 1141 NeuroDiderot, Inserm, Paris University, Paris, France; ^8^Medical Genomics Service for Rare Diseases, Necker-Sick children Hospital, AP-HP, Paris, France; ^9^Medical Genetics Laboratory, UMRS_1112, Alsace Medical Genetics Institute (IGMA), Strasbourg University and INSERM, Strasbourg, France; ^10^Medical Genetics Service, Alsace Medical Genetics Institute (IGMA), Strasbourg University Hospital, Strasbourg, France; ^11^Reference Center for Rare Disorders in Ophthalmic Genetics (CARGO), Filière SENSGENE, Strasbourg University Hospital, Strasbourg, France

## Abstract

Ciliopathies are rare genetic disorders caused by dysfunction of the primary or motile cilia. Their mode of inheritance is mostly autosomal recessive with biallelic pathogenic variants inherited from the parents. However, exceptions exist such as uniparental disomy (UPD) or the appearance of a *de novo* pathogenic variant in *trans* of an inherited pathogenic variant. These two genetic mechanisms are expected to be extremely rare, and few data are available in the literature, especially regarding ciliopathies. In this study, we investigated 940 individuals (812 families) with a suspected ciliopathy by Sanger sequencing, high-throughput sequencing and/or SNP array analysis and performed a literature review of UPD and *de novo* variants in ciliopathies. In a large cohort of 623 individuals (511 families) with a molecular diagnosis of ciliopathy (mainly Bardet-Biedl syndrome and Alström syndrome), we identified five UPD, revealing an inherited pathogenic variant and five pathogenic variants of *de novo* appearance (in *trans* of another pathogenic variant). Moreover, from these ten cases, we reported 15 different pathogenic variants of which five are novel. We demonstrated a relatively high prevalence of UPD and *de novo* variants in a large cohort of ciliopathies and highlighted the importance of identifying such rare genetic events, especially for genetic counseling.

## 1. Introduction

Ciliopathies are rare genetic disorders caused by a dysfunction of either the primary or motile cilia due to the loss of function of proteins implicated in ciliogenesis, protein trafficking to the cilium, or intraflagellar transport [1]. Covering more than 50 different clinical entities, ciliopathies share common phenotypic features but have variable expressivity. Bardet-Biedl syndrome (BBS, OMIM #209900), one of the most emblematic ciliopathies, is defined by *retinitis pigmentosa*, polydactyly, obesity, renal abnormalities, cognitive impairment, and hypogonadism [2]. To date, more than 24 genes have been implicated in BBS [3]. The Alström syndrome (AS, OMIM #203800) is a much rarer syndrome characterized by retinal dystrophy, obesity, neurosensory deafness, cardiomyopathy, and renal insufficiency [4] and is linked to a single gene *ALMS1* [5]. BBS and AS are both autosomal recessive (AR) diseases with, in general, a contribution of both parental alleles. However, this situation can be complicated by rare genetic events such as a uniparental disomy (UPD) or a variant of *de novo* appearance. UPD refers to the presence, in the same individual, of two chromosomes or part of a chromosome of the same pair inherited from only one of the parents [6]. Heterodisomy, corresponding to a pair of nonidentical chromosomes inherited from one parent and occurring at an earlier stage than meiosis I, can be distinguished from isodisomy, in which a single chromosome from one parent is duplicated and occurs at a later stage than meiosis II. Isodisomy is a rare event characterized by regions of homozygosity (ROHs) involving a single chromosome as opposed to consanguinity defined by large ROHs distributed on all chromosomes [7]. Classically, without pathological consequences for the carrier individual, this genetic mechanism can lead to the appearance of a disease when it occurs in imprinted regions or AR disorders. Similarly, the *de novo* occurrence of variants is a largely underestimated mode of inheritance in AR diseases. The frequency of these two mechanisms is anticipated to be very low, whereas few data are available for AR diseases.

In a large French cohort of patients with a suspected ciliopathy (*n* = 940), we analyzed 623 individuals with a definitive molecular diagnosis and highlighted a significant prevalence of UPD and *de novo* pathogenic variants in BBS and AS.

## 2. Materials and Methods

### 2.1. Editorial Policies and Ethical Considerations

The study protocol was approved by our Institutional Review Board “Comité de Protection des Personnes” (EST IV, N°DC-20142222) and complies with the Declaration of Helsinki. Written informed consent from all individuals and/or their parents was obtained.

### 2.2. Subjects

We studied 623 confirmed cases of ciliopathy from 940 individual samples sent to our reference diagnostic laboratory between 2002 and 2022. Among the 623 cases, 10 are of specific interest in this study, of which 2 were already reported by us: individual XV.30 [8] and I.25 [9]. In particular, individual XV.30 with a biallelic pathogenic variant in *IFT140* was reanalyzed, taking into account the parental segregation (not available at the time of the initial report).

### 2.3. Genetic Testing

Genetic analysis was performed on genomic DNA extracted from peripheral blood leukocytes, saliva, skin biopsy, chorionic villi, amniotic fluid, or fetal blood using the QIAamp DNA Mini kit (QIAGEN, Germany) according to the manufacturer's instructions. Multiple genetic investigations were performed on individuals as previously described [10] (Supplementary Table S1). Parental segregations of variants were confirmed using Sanger sequencing as previously described [10]. Uniparental disomy was confirmed by microsatellite marker analysis using either the PowerPlex 16 HS kit (Promega, USA) or gene-specific markers (supplementary Figure S3), and/or SNP array analysis using the Illumina Beadchip Array Human Cyto SNP-12 V2.1 and following the Infinium® HD Assay Ultra protocol (Illumina, USA). Microsatellite marker analyses also excluded errors in the identification of samples.

Genomic positions refer to the hg19/GRCh37 version of the human genome. Nomenclature of variants is in accordance with the following RefSeq [11] identifiers: *ALMS1*: NM_015120.4; *BBS1*: NM_024649.4; *BBS2*: NM_031885.3; *BBS4*: NM_033028.4; *BBS12*: NM_152618.2; and *IFT140*: NM_014714.3. Variants were classified according to the ACMG guidelines [12] (Supplementary Table S2).

### 2.4. Targeted High-Throughput Sequencing Panel

Briefly, exonic regions of DNA samples were captured with an in-solution enrichment methodology (Agilent QXT SureSelect custom panel) and sequenced with an Illumina NextSeq 550 instrument (paired-end sequencing, 2 × 150 bases, and 48 libraries per lane). Following alignment to the reference human genome (GRCh37/hg19) using BWA-MEM v0.7.5a [13], SNVs and indels were called with the HaplotypeCaller and UnifiedGenotyper from the Genome Analysis Toolkit v.3.4.46 [14] using our in-house pipeline (STARK) and following GATK best practices. Annotation and ranking of variants were performed by VaRank 1.4.3 [15] and Alamut Batch 1.11 (SOPHiA GENETICS); structural variants were detected using CANOES [16] and were annotated via AnnotSV 2.3 [17].

### 2.5. Genome Sequencing

For patient II.28, whole genome sequencing was performed on a NovaSeq 6000 (Illumina, USA) using paired-end sequencing. Reads were aligned onto the reference genome GRCh38.p13 using BWA-MEM (0.7.17) and variant calling was achieved with HaplotypeCaller by GATK (4.1.8.0) for the SNV/indels and with Manta (1.6.0) [18] and CNVnator (0.4.1) [19] for the structural variants. Annotation was performed using Variant Effect Predictor (version 98.3) [20].

### 2.6. Literature Review

A literature review was conducted using the PubMed in February 2021 with the following terms: “UPD AND recessive disorder”, “isodisomy AND recessive disorder”, “disomy AND recessive disorder”, and “disomy AND homozygote”. This led to the identification of 159 French-written or English-written articles, of which two were excluded based on the absence of primary data (number of cases, chromosome number, gene name, or parental origin of UPD). This resulted in 157 published full-text articles concerning about 174 cases that were included and reviewed by one coauthor (Supplementary Table S4).

## 3. Results

### 3.1. Description of the Cohort

Our cohort was composed of 940 individuals with suspected ciliopathies (AS *n* = 76, BBS *n* = 792, and other *n* = 72) from 812 families (AS *n* = 68, BBS *n* = 685, and other *n* = 59) (Supplementary data, Figure S1). Consanguinity was reported for 288 families (information was unavailable for 118 families). Of the 940 individuals, we identified biallelic pathogenic variants in 623 individuals from 511 families named “positive cases” (AS: *n* = 64 individuals from 57 families, BBS: *n* = 499 individuals from 406 families, and others: *n* = 60 individuals from 48 families; see supplementary Figure S2 and supplementary Table S3). Parental segregation was available for 60% of the positive cases (AS: *n* = 46 individuals from 39 families, BBS: *n* = 287 individuals from 224 families, and others: *n* = 31 individuals from 26 families), and, among them, consanguinity was reported for ~35% of families (AS: *n* = 16, BBS: *n* = 107, and others: *n* = 7). Moreover, 46.9% (30/64) and 61.5% (307/499) were homozygous for pathogenic variants in AS and BBS cohorts, respectively. We focused on 10 individuals (AS *n* = 5, BBS *n* = 4, and other *n* = 1) from 10 families harboring 15 different pathogenic variants of which five are novel (Supplementary Table S2). Individuals with AS presented with typical clinical features including retinal dystrophy (4/5), obesity (4/5), hearing loss (4/5), and cardiomyopathy (3/5) (Table 1). Individual II.28 had a unilateral bifid thumb, a micropenis, and a 13th pair of ribs in addition to typical criteria for AS. Regarding the four BBS individuals, individuals XXX.28 and XVIII.23 fulfilled the BBS clinical criteria (*retinitis pigmentosa*, polydactyly, obesity, and cognitive impairment), while the other two only had a few clinical manifestations: one was a fetus with hyperechogenic kidneys, postaxial polydactyly, and fetal growth parameters above the 97th percentile, and the last one had hyperechogenic kidneys, postaxial polydactyly, and psychomotor delay during infancy (Table 1). Individual XV.30 carried a homozygous duplication of exons 27 to 30 (c.3454-488_4182+2588dup, p.Tyr1152_Thr1394dup) in the *IFT140* gene, resulting in a Saldino-Mainzer syndrome. This 21-year-old woman had *retinitis pigmentosa*, short stature, brachydactyly, and tubulointerstitial nephritis that required renal transplant (Table 1). Eight of these 10 individuals carried truncating variants. The recurrent *BBS1* p.Met390Arg [21] variation was found in one individual, as well as a nucleotide change in the *BBS4* gene that was predicted to be a missense (c.220G > A, p.(Ala74Thr)) but resulted in a splice alteration. Indeed, analysis of the patient's RNA revealed a deletion of exons 5 and 6 (r.221_405del) of the *BBS4* mRNA leading to a premature STOP codon (p.(Ala74Glyfs^∗^5)) (data not shown).

### 3.2. Identification of Five UPD in Positive Cases and Literature Review

**Figure 1:**
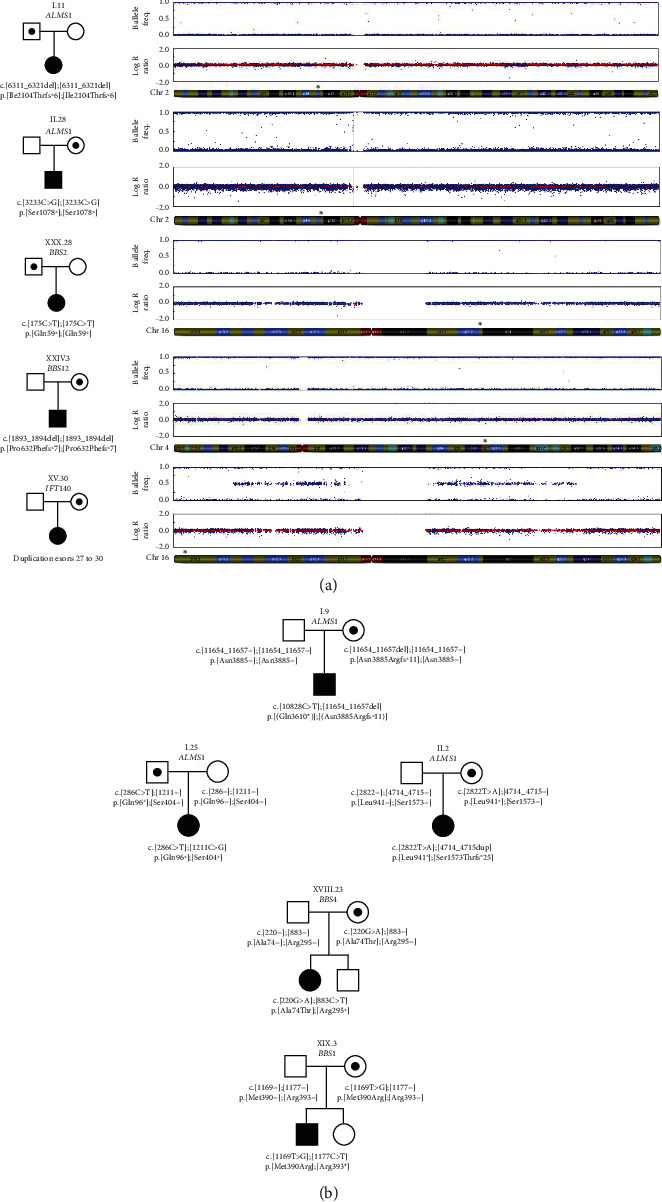
Identification of five uniparental isodisomies (iUPD) and five *de novo* variants among 623 positive cases for ciliopathies genes. (a) Pedigrees and SNP array profiles of the five iUPD implicated in AS (*ALMS1*), BBS (*BBS2* and *BBS12*), and Mainzer-Saldino syndrome (*IFT140*). The SNP array data is separated into two panels for each patient. The upper panel shows the B-allele frequency representing the variant status (an allelic composition measurement) for each SNP position. Heterozygous SNP are in the middle (~0.5), while homozygous are at either 0 or 1. This can be used to highlight the loss of heterozygosity regions for all the cases except XV.30. The lower panel shows the log *R* ratio to identify copy number variants (CNV). Above each chromosome map, a “^∗^” indicates the gene position. (b) Pedigrees of the five individuals carrying a *de novo* variant implicated in AS (*ALMS1*) and BBS (*BBS1* and *BBS4*).

Among the positive cases, we used SNP arrays to confirm ROHs involving only one chromosome in five cases, corresponding to complete (4/5) or segmental (1/5) uniparental isodisomy (iUPD). These iUPD revealed a pathogenic variant in AR genes (2x*ALMS1*, 1x*BBS2*, 1x*BBS12,* and 1x*IFT140*) (Table 1). The implicated variants were all truncating variants. The iUPD involved chromosomes 2, 4, and 16 (Figure 1(a)), and the parental origin of the chromosome was maternal in three cases and paternal in two cases. Parental age at conception ranged from 23 years to 39 years (Table 1). The iUPD represented, respectively, ~3% (2/64) and 0.4% (2/499) of AS and BBS patients in our cohort. These rates increased to ~4% (2/46) and~0.7% (2/287) if we only considered nonconsanguineous families with parental segregation available. Undoubtedly, parental segregation represents important information to diagnose potential UPD. In cases where parental segregation was not available, we selected 26 individuals carrying homozygous rare variants for whom no consanguinity was reported and performed SNP array analysis to look for ROHs involving a whole chromosome. This analysis revealed large regions of ROHs suggesting consanguinity in nine out of these 26 individuals but did not identify additional iUPD.

To understand how our cases are associated with other UPD cases, we reviewed 157 publications describing UPD cases in autosomal recessive disorders. We found 161 cases affecting 133 different genes (Supplementary Table S4). The majority of the genes (*n* = 109) were isolated cases implicating a single individual. The remaining genes (*n* = 24) had 2 or more cases and a maximum of 4. In our review, chromosomes 1, 2, 6, and 16 were the most reported ones, while no cases could be found on chromosomes 18, 19, and 21 (Supplementary Table S5). Interestingly, the frequencies of paternal and maternal origin are equivalent (74 paternal and 79 maternal).

### 3.3. Identification of Five *De Novo* Variants in Positive Cases

Considering *de novo* variants in our cohort of positive cases with assessed segregation and paternity/maternity, we identified five truncating variants in *trans* of an inherited pathogenic variant (AS: *n* = 3 and BBS: *n* = 2) (Figure 1(b) and Table 1). The pathogenic *de novo* variants appeared on the paternal allele in four individuals, and the parental age at conception ranged from 29 to 45 years. Parental segregation by Sanger sequencing showed no evidence of mosaicism (albeit a minimum of 15%) on blood samples from the parents. Finally, a *de novo* event is involved in ~4.7% (3/64) to 6.5% (3/46) and 0.4% (2/499) to 0.7% (2/287) of AS and BBS individuals, respectively, depending on parental segregation availability.

## 4. Discussion

In this study, we investigated 623 confirmed cases of ciliopathies including 64 AS, 499 BBS, and 60 individuals with another ciliopathy-related disorder. We identified five iUPD (AS: *n* = 2, BBS: *n* = 2, and *IFT140:n* = 1) and five *de novo* variants (AS *n* = 3 and BBS *n* = 2), representing 1.6% (10/623) of our cohort (Supplementary Figure S1).

UPD is a well-known genetic mechanism, but its prevalence is difficult to assess. Recent studies based on SNP array genotyping or trio-based clinical exomes in a pathologic context revealed a rate of 1-3 UPD per 1,000 individuals [7, 22]. In the general population, Nakka et al. estimated a UPD prevalence of 1 per 2,000 births from genome-wide SNP analyses of 214,915 healthy trios [23]. These studies highlighted a greater number of UPD depending of the chromosome involved and its parental origin, reinforcing previous findings [22, 23]. As expected, there is an overrepresentation of chromosome 16 UPD compared to other chromosomes due to a possible trisomy rescue [24], and the maternal origin of the chromosome is a major risk of UPD appearance. Remarkably, in our study, two out of five UPD involved the chromosome 16, and three were from maternal origin (Table 1). In our literature review of a total of 161 articles, only a single ciliopathy case was reported in the *IFT140* gene [25], and none in any BBS genes or the *ALMS1* gene. However, a UPD (16) was reported to be the cause of Bardet-Biedl syndrome 4 in one individual in another large study [7]. As chromosomes 1, 4, 14, 15, 16, and 21 are often implicated in UPD [22, 23] and carry a BBS gene, this information should be taken into account when analyzing a trio for BBS with inheritance discrepancy. It should be noted that specific bioinformatics pipelines can be applied to detect such cases [26]. Furthermore, although UPD revealed another pathogenic variant for an AR disease in our patients, we did not systemically sequence other overlapping disease genes as there was no evidence for a second disorder.

Another rarely reported event in AR diseases is the presence of a pathogenic variant of *de novo* occurrence in *trans* of an inherited pathogenic variant. *De novo* variants are the main etiology of some autosomal dominant disorders such as intellectual disability; however, they are less reported in AR diseases. For instance, Retterer et al. [27] and Yang et al. [28], respectively, analyzed 3,000 and 2,000 individuals using whole exome sequencing and did not report a single *de novo* variant in AR disorders, although they represented around one third of their cohorts. It has been demonstrated that increased parental age and paternal origin are risk factors for *de novo* occurrence [29, 30]. The paternal origin of the *de novo* variant was observed in four individuals of our cohort. Nevertheless, given the limited number of cases, we could not presume any result on parental age regarding these variants.

Knowing the molecular mechanism of the disease has a direct impact on the genetic counselling delivered to the couple. In the case of UPD and *de novo* variants in an AR disease, the risk of recurrence of the disease drastically drops from 25% to ~1% per pregnancy [31]. However, in the case of *de novo* variants, the possibility of a germline mosaicism that, in certain cases, could reach a high percentage [32] should not be overlooked. In our samples, we were only able to exclude this on a single tissue (blood sample) and using a moderately sensitive technique (Sanger).

The identification of UPD and *de novo* variants requires careful attention. Indeed, these events can be masked by trioanalysis in AR disorder if one only considers variants inherited from both parents. Some clues can help the analysis, such as unmatched parental segregation or the presence of a rare variant at the homozygous state without consanguinity. The systematic estimation of the frequency of UPD and *de novo* variants in more AR disorders should be performed.

Limitations in our study include a lack of segregation in 40% of the cases (*n* = 259 individuals). Although segregation could be achieved in 365 individuals from 290 families, it is sometimes difficult to have access to other family members. Concerning *de novo* variations, mosaicism was only partially assessed and could have been evaluated on other tissues such as sperm for four cases (male origin). SNP arrays were not performed systematically to evaluate consanguinity involvement in homozygous cases.

In conclusion, this study allowed an evaluation of rare inheritance patterns in two rare ciliopathies (e.g., AS and BBS) and highlighted a large number of UPD and *de novo* variants.

## Figures and Tables

**Table 1:** Genotype and phenotype data of individuals carrying a uniparental disomy or a *de novo* variant.

Family ID	UPD/*de novo*	Locus gene	Paternal allele	Maternal allele	CMA nomenclature according to ISCN 2020	Inheritance (age at conception)	Age at last examination	RP	P	O	K	ID	C	D	Other
I.11_ALMS	UPD	2p13.1 *ALMS1*	c.6311_6321del, p.(Ile2104Thrfs^∗^6)		arr(2)x2 hmz pat	Pat. (32 y)	6 y	+	—	+	—	—	+	+	

II.28_ALMS	UPD	2p13.1 *ALMS1*		c.3233C>G, p.(Ser1078^∗^)	arr(2)x2 hmz mat	Mat. (39 y)	2 y	—	+	+	—	—	+	—	Micropenis 13th pair of ribs

XXX.28	UPD	16q13 *BBS2*	c.175C>T, p.(Gln59^∗^)		arr(16)x2 hmz pat	Pat. (34 y)	18 y	+	+	+	+	+	—	—	

XXIV.3	UPD	4q27 *BBS12*		c.1893_1894del, p.(Pro632Phefs^∗^7)	arr(4)x2 hmz mat	Mat. (34 y)	2 y	—	+	—	+	+	—	—	

XV.30	UPD	16p13.3 *IFT140*		c.3454-488_4182+2588dup, p.Tyr1152_Thr1394dup	arr[GRCh37] 16p13.3p13.13(105320_11030742)x2 hmz mat,16q23.1q24.3(74735148_90148796)x2 hmz mat	Mat. (23 y)	50 y	+	—	+	+	—	—	—	Short stature, brachydactyly, and tubulointerstitial nephritis

I.9_ALMS	*de novo*	2p13.1 *ALMS1*	**c.10828C>T, p.(Gln3610** ^∗^ **)**	c.11654_11657del, p.(Asn3885Argfs^∗^11)		Pat. (45 y)	17 y	+	—	+	—	—	—	+	Hypothyroidism

I.25_ALMS	*de novo*	2p13.1 *ALMS1*	c.286C>T, p.(Gln96^∗^)	c.1211C>G, p.(Ser404^∗^)		Mat. (32 y)	14 y	+	—	—	—	—	+	+	

II.2_ALMS	*de novo*	2p13.1 *ALMS1*	**c.4714_4715dup, p.(Ser1573Thrfs** ^∗^ **25)**	c.2822T>A, p.(Leu941^∗^)		Pat. (29 y)	4 y	+	—	+	—	+	—	+	

XIX.3	*de novo*	11q13.2 *BBS1*	**c.1177C>T, p.(Arg393** ^∗^ **)**	c.1169T>G, p.(Met390Arg)		Pat. (31 y)	Fetus	—	+	+†	+	—	—	—	

XVIII.23	*de novo*	15q24.1 *BBS4*	**c.883C>T, p.(Arg295** ^∗^ **)**	c.220G>A, p.(Ala74Thr)		Pat. (28 y)	26 y	+	+	+	—	+	—	—	

*De novo* variants are in bold. †Growth parameters >95*e* percentile. Abbreviations: CMA: chromosomal microarray; Pat.: paternal; Mat.: maternal; y: years; RP: *retinitis pigmentosa*; P: polydactyly; O: obesity; K: kidney anomaly; ID: intellectual disability; C: cardiomyopathy; D: deafness.

## Data Availability

Data generated or analyzed during this study are included in the published article and the corresponding online supporting Information. All variants have been submitted to ClinVar using the following range of accession numbers SCV002559820-SCV002559831 (https://www.ncbi.nlm.nih.gov/clinvar/).

## Supplementary Materials

Supplementary Materials Supplementary data includes the following figures and tables. Figure S1: flowchart illustrating the distribution of positive cases among the cohort. Figure S2: pie chart illustrating the distribution of the 499 BBS-positive cases and their genes in the cohort. Figure S3: microsatellite analysis for 3 families, including 2 UPD (A and B) and 1 *de novo* (C). Table S1: analyses performed on individuals carrying an UPD or a *de novo* variant. Table S2: list of variations identified and their ACMG classification. Table S3: list of pathologies and related genes identified in positive cases in the category named “other” together with detailed clinical data. Table S4: literature review raw data including article title, DOI/PMID, gene and chromosome, parental origin, and the disease. Table S5: distribution of the UPD cases in the literature per chromosome.
