# Supplementary material for: Unexpected Inheritance Patterns in a Large Cohort of Patients with a Suspected Ciliopathy
Source: Hum Mutat. 2023 Aug 9;2023:2564200. doi: 10.1155/2023/2564200 (PMC11918889; doi:10.1155/2023/2564200)
Supplement: Supplementary Materials — Supplementary data includes the following figures and tables. Figure S1: flowchart illustrating the distribution of positive cases among the cohort. Figure S2: pie chart illustrating the distribution of the 499 BBS-positive cases and their genes in the cohort. Figure S3: microsatellite analysis for 3 families, including 2 UPD (A and B) and 1 de novo (C). Table S1: analyses performed on individuals carrying an UPD or a de novo variant. Table S2: list of variations identified and their ACMG classification. Table S3: list of pathologies and related genes identified in positive cases in the category named “other” together with detailed clinical data. Table S4: literature review raw data including article title, DOI/PMID, gene and chromosome, parental origin, and the disease. Table S5: distribution of the UPD cases in the literature per chromosome. [file 2564200.f1.zip › BBS_UnexpectedTransmission_SuppData_HM_Final (1).docx]

**Supplementary data**

**
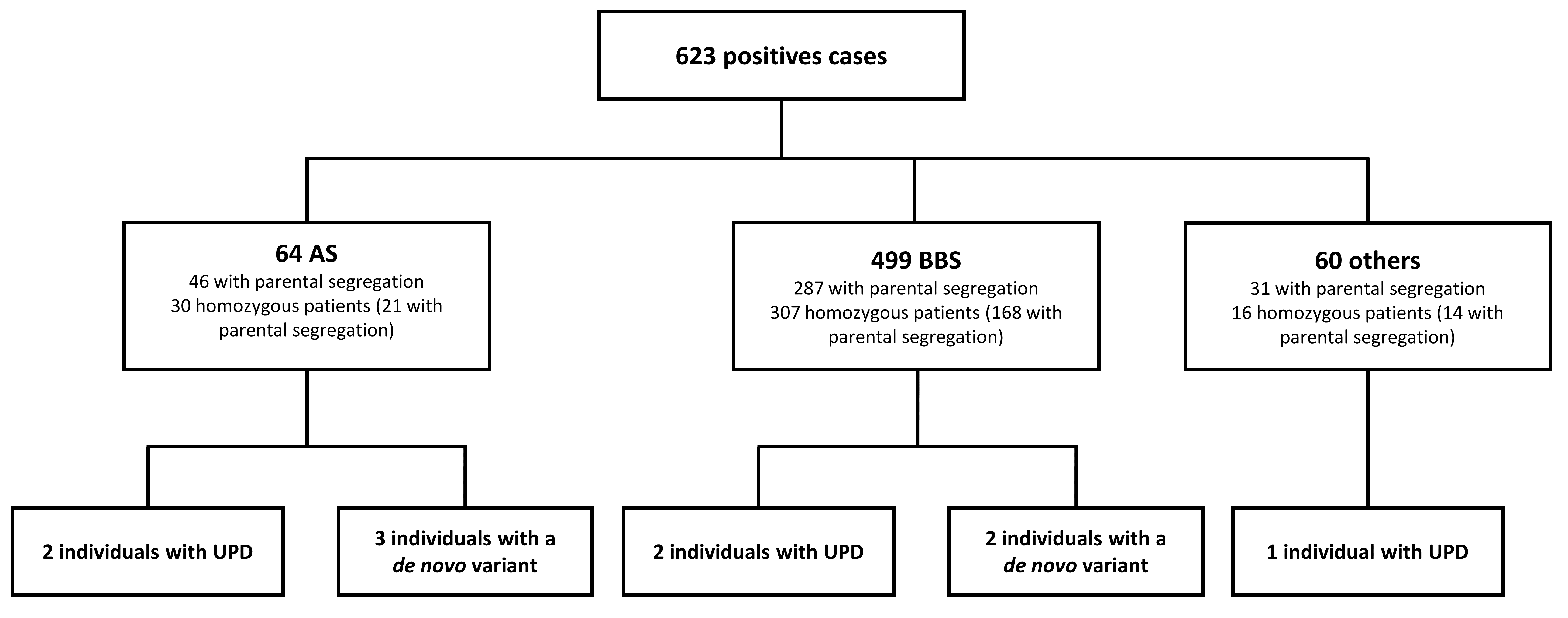
**

**Figure S1 : Flowchart illustrating the distribution of positive cases in the cohort.** AS: Alström syndrome; BBS: Bardet-Biedl syndrome; UPD: uniparental disomy.

**
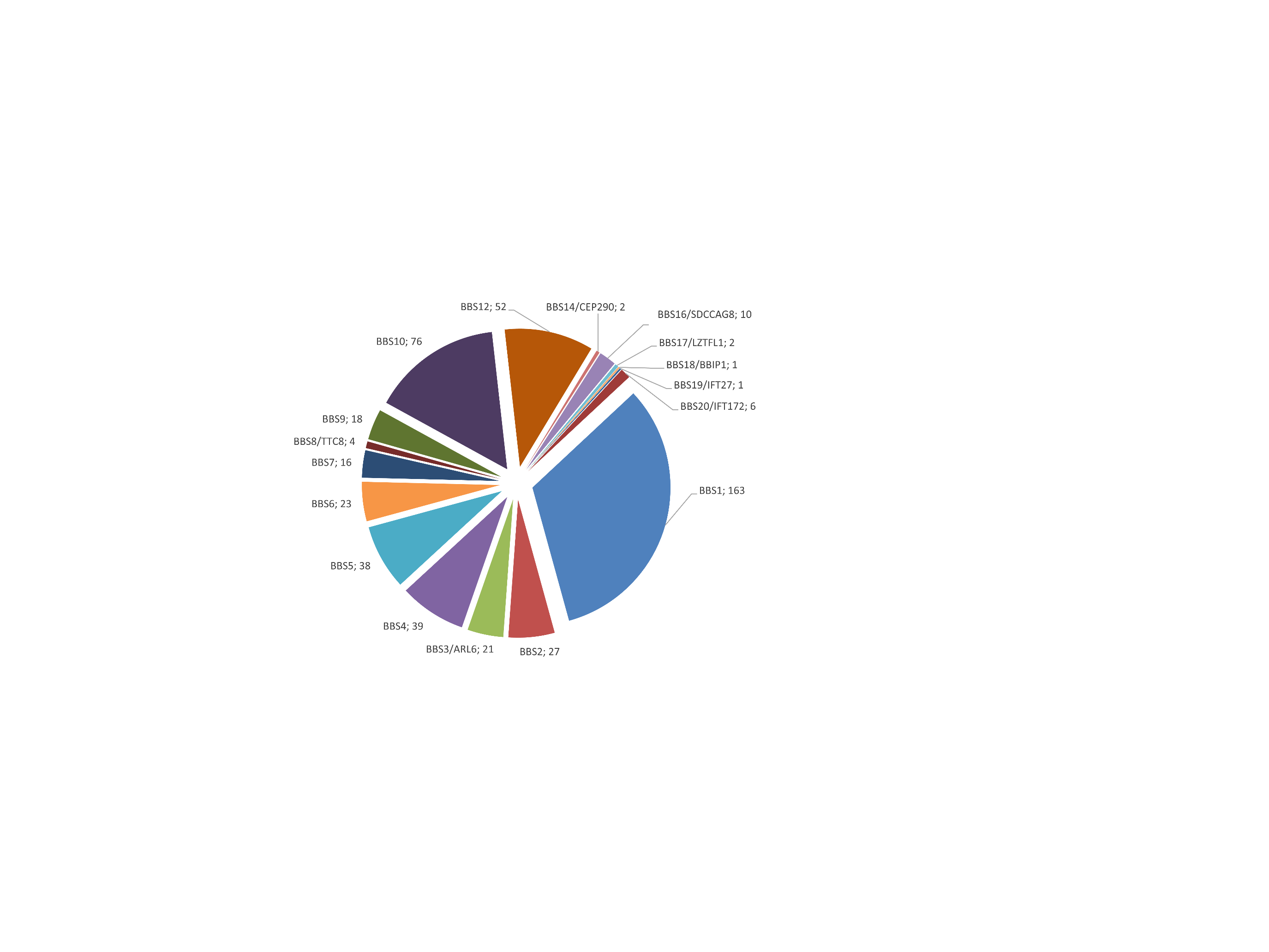
**

**Figure S2 : Pie chart illustrating the distribution of the 499 BBS positive cases and their genes in the cohort.**

**
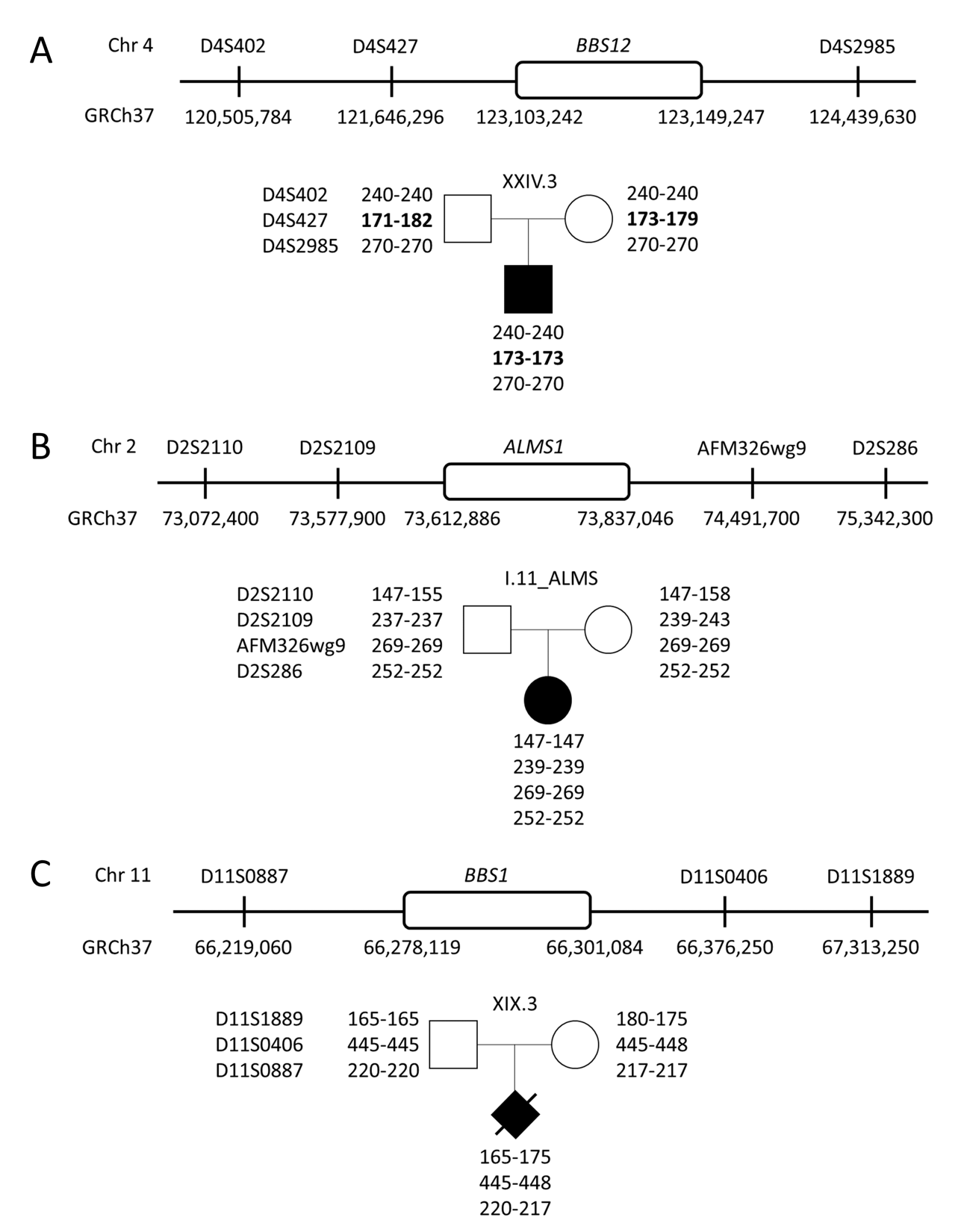
**

**Figure S3 : Microsatellite analysis for 3 families, including 2 UPD (A and B) and 1 *de novo* (C).**

**Table S1: Analyses performed on individuals carrying an UPD or a *de novo* variant.** The 18 genes panel version includes the *BBS1-BBS17* and *ALMS1* genes.The 58 genes panel version includes the *BBS1-BBS20*, *NPHP1-19*, *MKS1-17*, *JBTS1-22*, *SLSN1-8* and *ALMS1*. Abbreviations: BBS: Bardet-Biedl syndrome, HTS: high throughput sequencing, NPHP: nephronophthisis, MKS: Meckel syndrome, JBTS: Joubert syndrome, SLSN: Senior Løken.

| **Individual** | **Analyses** |
| --- | --- |
| I.11 (ALMS) | HTS 58 genes, microsatellite |
| II.28 (ALMS) | Genome sequencing |
| XXX.28 | Sequencing of the *BBS1,* *BBS2, BBS7, BBS8, BBS10,* and *BBS12* genes |
| XXIV.3 | HTS 58 genes, microsatellite |
| XV.30 | HTS Ciliome (Geoffroy et al., 2018a) |
| I.9 (ALMS) | HTS 30 genes (Redin et al., 2012) |
| I.25 (ALMS) | HTS 18 genes (Mauring et al., 2020a) |
| II.2 (ALMS) | HTS 30 genes (Redin et al., 2012) |
| XIX.3 | HTS 30 genes (Redin et al., 2012), microsatellite |
| XVIII.23 | Sequencing of the *BBS1-12* genes |

**Table S2: List of variations identified and their ACMG classification.** Variant effect on the nearest splice site was predicted using MaxEntScan (MES), NNSplice (NNS) and Splice Site Finder (SSF) (Shapiro and Senapathy, 1987; Reese et al., 1997; Yeo and Burge, 2004) by calculating score change between the wild type and the mutated sequences expressed as percent differences. Missense effect was assessed by PolyPhen-2 (Humdiv/Humvar) and SIFT (Kumar et al., 2009; Adzhubei et al., 2010). Nomenclature of variants is in accordance with the following RefSeq (O’Leary et al., 2015) identifiers: *ALMS1*: NM_015120.4, *BBS1*: NM_024649.4, *BBS2*: NM_031885.3, *BBS4*: NM_033028.4, *BBS12*: NM_152618.2, *IFT140*: NM_014714.3.

| **Gene** | **Localisation** | **cDNA (c.), Protein (p.)** | **Type** | **ACMG criteria** | **ACMG Class** | **PolyPhen-2** | **SIFT** | **SSF** | **MES** | **NNS** | **Reference** |
| --- | --- | --- | --- | --- | --- | --- | --- | --- | --- | --- | --- |
| *ALMS1* | 2p13.1 | c.286C>T, p.(Gln96*) | Nonsense | PVS1, PM2, PP3 | 5 |  |  |  |  |  | (Mauring et al., 2020b) |
| *ALMS1* | 2p13.1 | c.1211C>G, p.(Ser404*) | Nonsense | PVS1, PS2, PM2, PP3 | 5 |  |  |  |  |  | (Mauring et al., 2020b) |
| *ALMS1* | 2p13.1 | c.2822T>A, p.(Leu941*) | Nonsense | PVS1, PM2, PP3 | 5 |  |  |  |  |  | (Marshall et al., 2015) |
| *ALMS1* | 2p13.1 | c.3233C>G, p.(Ser1078*) | Nonsense | PVS1, PM2, PP3 | 5 |  |  |  |  |  |  |
| *ALMS1* | 2p13.1 | c.4714_4715dup, p.(Ser1573Thrfs*25) | Frameshift | PVS1, PS2, PM2, PP3 | 5 |  |  |  |  |  | (Marshall et al., 2015) |
| *ALMS1* | 2p13.1 | c.6311_6321del, p.(Ile2104Thrfs*6) | Frameshift | PVS1, PM2, PP3 | 5 |  |  |  |  |  |  |
| *ALMS1* | 2p13.1 | c.10828C>T, p.(Gln3610*) | Nonsense | PVS1, PS2, PM2, PP3 | 5 |  |  |  |  |  | (Marshall et al., 2015) |
| *ALMS1* | 2p13.1 | c.11654_11657del, p.(Asn3885Argfs*11) | Frameshift | PVS1, PM2, PP3 | 5 |  |  |  |  |  |  |
| *BBS1* | 11q13.2 | c.1169T>G, p.(Met390Arg) | Missense | PVS1, PM2, PP3 | 5 |  |  |  |  |  |  |
| *BBS1* | 11q13.2 | c.1177C>T, p.(Arg393*) | Nonsense | PVS1, PM2, PP3 | 5 |  |  |  |  |  | (Mary et al., 2019) |
| *BBS2* | 16q13 | c.175C>T, p.(Gln59*) | Nonsense | PVS1, PM2, PP3 | 5 |  |  |  |  |  | (Katsanis et al., 2001) |
| *BBS4* | 15q24.1 | c.220G>A, p.(Ala74Thr) | Missense  (splice effect) | PS3, PM2, PM3, PP3 | 5 | Probably damaging  (0.999/0.954) | Deleterious  (0.00) | -12.9% | -34.6% | -4.3% |  |
| *BBS4* | 15q24.1 | c.883C>T, p.(Arg295*) | Nonsense | PVS1, PS2, PM2, PP3 | 5 |  |  |  |  |  | (Weisschuh et al., 2020) |
| *BBS12* | 4q27 | c.1893_1894del, p.(Pro632Phefs*7) | Frameshift | PVS1, PM2, PP3 | 5 |  |  |  |  |  | (Stoetzel et al., 2007) |
| *IFT140* | 16p13.3 | c.3454-488_4182+2588dup, p.Tyr1152_Thr1394dup  (duplication of exon 27 to 30) | Copy Number Variant | PVS1, PM2, PP3 | 5 |  |  |  |  |  | (Geoffroy et al., 2018b) |

**Table S3: List of pathologies and related genes identified in positive cases in the category named “other” together with detailed clinical data.**

See SuppTable3_OtherDiseases.xlsx file.

**Table S4: Literature review raw data including article title, DOI/PMID, gene and chromosome, parental origin and the disease.**

| **Article title** | **DOI/PMID** | **Article Type** | **Chr** | **Gene** | **Parental Origin** | **Pathology** |
| --- | --- | --- | --- | --- | --- | --- |
| Segmental Maternal UPD of Chromosome 7q in a Patient With Pendred and Silver Russell Syndromes-Like Features Valentina Cirello | 10.3389/fgene.2018.00600 | Case report | 7 | *SLC26A4* | mat | Pendred + SRS |
| Uniparental isodisomy caused autosomal recessive diseases: NGS-based analysis allows the concurrent detection of homogenous variants and copy-neutral loss of heterozygosity Bing Xiao | https://doi.org/10.1002/mgg3.945 | Case report (2 cases) | 1 | *AGL* | pat | Glycogen storage disease type III |
|  |  |  | 9 | *SURF1* | UPiD9pat + UPiD10mat | Leigh syndrome |
| Congenital ichthyosis in Prader-Willi syndrome associated with maternal chromosome 15 uniparental disomy: Case report and review of autosomal recessive conditions unmasked by UPD Karthik Muthusamy | https://doi.org/10.1002/ajmg.a.61792 | Case report + review DUP15mat avec PWS | 15 | *CERS3* | mat | Congenital Ichthyosis + PWS |
| Segmental and total uniparental isodisomy (UPiD) as a disease mechanism in autosomal recessive lysosomal disorders: evidence from SNP arrays Ineke Labrijn-Marks | https://doi.org/10.1038/s41431-019-0348-y | Case report (5 cases) | 4 | *IDUA* | mat | Mucopolysaccharidose type I |
|  |  |  | 17 | *GAA* | mat | Pompe disease |
|  |  |  | 17 | *GAA* | mat | Pompe disease |
|  |  |  | 17 | *GAA* | mat | Pompe disease |
|  |  |  | 17 | *GAA* | pat | Pompe disease |
| Uniparental disomy of chromosome 8 leading to homozygosity of a CYP11B1 mutation in a patient with congenital adrenal hyperplasia: implication for a rare etiology of an autosomal recessive disorder Keiko Matsubara | https://doi.org/10.1507/endocrj.ej13-0509 | Case report | 8 | *CYP11B1* | pat | Adrenal hyperplasia, congenital, due to 11-beta-hydroxylase deficiency |
| Maternal uniparental isodisomy causing autosomal recessive GM1 gangliosidosis: a clinical report Jessica E King | https://doi.org/10.1007/s10897-014-9720-9 | Case report | 3 | *GLB1* | mat | GM1 gangliosidosis |
| Uniparental disomy as an unexpected cause of Meckel-Gruber syndrome: report of a case Nadia Ortiz Bruechle | https://doi.org/10.1007/s00467-017-3710-8 | Review of a case | 8 | *MKS3* | mat | Meckel-Gruber syndrome |
| Uniparental disomy causes deficiencies of vitamin K-dependent proteins M A Dasi | https://doi.org/10.1111/jth.13517 | Case report | 2 | *GGCX* | pat | Deficiency of all vitamin K-dependent coagulant factors (VKCFD) |
| Perinatal hypophosphatasia caused by uniparental isodisomy Atsushi Watanabe | 10.1016/j.bone.2013.12.009 | Case report | 1 | *ALPL* | pat | Hypophosphatasia |
| PMM2-CDG caused by uniparental disomy: Case report and literature review Laurien Vaes | 10.1002/jmd2.12122 | Case report (7 cases) | 16 | *PMM2* | mat ihUPD16 | Phosphomannomutase 2 deficiency |
| CDG-Id caused by homozygosity for an ALG3 mutation due to segmental maternal isodisomy UPD3(q21.3-qter) E Schollen | 10.1016/j.ejmg.2005.01.002 | Case report | 3 | *ALG3* | mat | CDG-Id |
| Congenital myasthenic syndrome caused by a frameshift insertion mutation in GFPT1 Szabolcs Szelinger | 10.1212/NXG.0000000000000468 | Case report | 2 | *GFPT1* | mat | Congenital myasthenic syndrome |
| Segmental uniparental disomy leading to homozygosity for a pathogenic mutation in three recessive metabolic diseases Belén Pérez | 10.1016/j.ymgme.2011.10.019 | Letter to the editor | 13 | *PCCA* | mat | Propionic acidemia |
|  |  |  | 5 | *MTRR* | pat | Homocystinuria cblE type |
|  |  |  | 16 | *PMM2* | pat | Congenital defect in glycosylation due to phosphomannomutase 2 deficiency (PMM2-CDG) |
| Uniparental isodisomy of chromosome 1 results in glycogen storage disease type III with profound growth retardation Emanuela Ponzi | 10.1002/mgg3.634 | Case report | 1 | *AGL* | pat | Glycogen storage disease type III |
| Adrenal Insufficiency, Sex Reversal, and Angelman Syndrome due to Uniparental Disomy Unmasking a Mutation in CYP11A1 Ahlee Kim | 10.1159/000487638 | Case report | 15 | *CYP11A1* | pat | Cholesterol side-chain cleavage enzyme (P450scc) deficiency + SA |
| Warburg Micro Syndrome 1 due to Segmental Paternal Uniparental Isodisomy of Chromosome 2 Detected by Whole-Exome Sequencing and Homozygosity Mapping Abdullah Sezer | 10.1159/000509214 | Case report | 2 | *RAB3GAP1* | pat | Warburg Micro Syndrome 1 |
| Obesity and developmental delay in a patient with uniparental disomy of chromosome 2 T Yu | 10.1038/ijo.2016.160 | Case report | 2 | *GPBAR1/CAPN10* | mat | Obesity and developmental delay |
| Rhizomelic chrondrodysplasia punctata type 2 resulting from paternal isodisomy of chromosome 1 Graeme Nimmo | 10.1002/ajmg.a.33489 | Case report | 1 | *GNPAT* | pat | Rhizomelic chrondrodysplasia punctata type 2 |
| Maternal uniparental disomy 14 revealed by alpha 1 antitrypsin deficiency Noémie Laverdure | 10.1016/j.clinre.2014.01.011 | Case report (2 cases) | 14 | *SERPINA1* | mat | Alpha 1 antitrypsin deficiency +/- PWS |
|  |  |  | 14 | *SERPINA1* | mat | Alpha 1 antitrypsin deficiency +/- PWS |
| Uniparental disomy of chromosome 16 unmasks recessive mutations of FA2H/SPG35 in 4 families Anne S Soehn | 10.1212/WNL.0000000000002843 | Case report (4 cases) | 16 | *FA2H* | mat | Spastic paraplegia type 35 |
|  |  |  | 16 | *FA2H* | pat | Spastic paraplegia type 35 |
|  |  |  | 16 | *FA2H* | pat | Spastic paraplegia type 35 |
|  |  |  | 16 | *FA2H* | pat | Spastic paraplegia type 35 |
| Detection of Hereditary 1,25-Hydroxyvitamin D-Resistant Rickets Caused by Uniparental Disomy of Chromosome 12 Using Genome-Wide Single Nucleotide Polymorphism Array Mayuko Tamura | 10.1371/journal.pone.0131157 | Case report | 12 | *VDR* | mat | Hereditary 1,25-Hydroxyvitamin D-Resistant Rickets |
| An unexpected transmission of von Willebrand disease type 3: the first case of maternal uniparental disomy 12 Pierre Boisseau | 10.3324/haematol.2010.036897 | Case report | 12 | *VWF* | mat | von Willebrand disease type 3 |
| Maternal uniparental isodisomy and heterodisomy on chromosome 6 encompassing a CUL7 gene mutation causing 3M syndrome K Sasaki | 10.1111/j.1399-0004.2010.01599.x | Case report | 6 | *CUL7* | ihUPD6mat | 3M syndrome |
| Fatal malonyl CoA decarboxylase deficiency due to maternal uniparental isodisomy of the telomeric end of chromosome 16 S Malvagia | 10.1111/j.1469-1809.2007.00373.x | Case report | 16 | *MLYCD* | mat | Malonic aciduria |
| An ABCC8 gene mutation and mosaic uniparental isodisomy resulting in atypical diffuse congenital hyperinsulinism Khalid Hussain | 10.2337/db07-0998 | Case report | 11 | *ABCC8* | pat | Atypical diffuse congenital hyperinsulinism |
| Maternal uniparental disomy of chromosome 4 in a patient with limb-girdle muscular dystrophy 2E confirmed by SNP array technology C E Cottrell | 10.1111/j.1399-0004.2011.01681.x | Case report | 4 | *SGCB* | mat | Limb-girdle muscular dystrophies |
| Uniparental disomy of chromosome 2 resulting in lethal trifunctional protein deficiency due to homozygous alpha-subunit mutations Ute Spiekerkoetter | 10.1002/humu.10142 | Case report (2 cases) | 2 | *HADHA* | mat | Trifunctional protein deficiency |
|  |  |  | 2 | *HADHA* | mat | Trifunctional protein deficiency |
| Microarray analysis unmasked paternal uniparental disomy of chromosome 12 in a patient with isolated sulfite oxidase deficiency Sun Young Cho | 10.1016/j.cca.2013.08.013 | Case report | 12 | *SUOX* | pat | Sulfite oxidase deficiency |
| Donnai-Barrow syndrome (DBS/FOAR) in a child with a homozygous LRP2 mutation due to complete chromosome 2 paternal isodisomy Sibel Kantarci | 10.1002/ajmg.a.32381 | Case report | 2 | *LRP2* | pat | Donnai-Barrow |
| Maternal Isodisomy for Chromosome 2p Causing Severe Congenital Hypothyroidism Bert Bakker | 10.1210/jcem.86.3.7313 |  | 2 | *TPO* | mat | Congenital hypothyroidism |
| Retinal dystrophy due to paternal isodisomy for chromosome 1 or chromosome 2, with homoallelism for mutations in RPE65 or MERTK, respectively Debra A Thompson | 10.1086/338455 | Case report | 1 | *RPE65* | pat | Retinal dystrophy |
| Retinal dystrophy due to paternal isodisomy for chromosome 1 or chromosome 2, with homoallelism for mutations in RPE65 or MERTK, respectively Debra A Thompson | 10.1086/338455 | Case report | 2 | *MERTK* | pat | Retinal dystrophy |
| Sickle cell disease resulting from uniparental disomy in a child who inherited sickle cell trait Jeffrey J Swensen | 10.1182/blood-2010-05-284331 | Case report | 11 | *HBB* | pat | Sickle cell disease |
| Uniparental disomy: a novel mechanism for thalassemia major. Blood. 1992;80(1):287-289. | 10.1182/blood.V80.1.287.287 | Letter to the editor | 11 | *HBB* | pat | Major beta-thalassemia + BWS |
| Morquio A syndrome due to maternal uniparental isodisomy of the telomeric end of chromosome 16 S Catarzi | 10.1016/j.ymgme.2011.11.196 | Case report | 16 | *GALNS* | mat | Morquio A |
| Complete paternal uniparental isodisomy for chromosome 1 revealed by mutation analyses of the TRKA (NTRK1) gene encoding a receptor tyrosine kinase for nerve growth factor in a patient with congenital insensitivity to pain with anhidrosis Y Miura | 10.1007/s004390000369 | Case report | 1 | *TRKA* | pat | Congenital anhidrosis |
| Zellweger syndrome resulting from maternal isodisomy of chromosome 1 Claire L S Turner | 10.1002/ajmg.a.31912 | Case report | 1 | *PEX* | mat | Zellweger |
| Fumarase deficiency caused by homozygous P131R mutation and paternal partial isodisomy of chromosome 1 Wen-Qi Zeng | 10.1002/ajmg.a.31186 | Case report | 1 | *P131R* | pat | Fumarase deficiency |
| Complete paternal isodisomy for chromosome 8 unmasked by lipoprotein lipase deficiency P Benlian | PMID: 8755931 | Case report | 8 | *LPL* | pat | Lipoprotein lipase deficiency |
| Partial paternal uniparental disomy (UPD) of chromosome 1 in a patient with Stargardt disease | PMID: 17277736 | Case report | 1 | *ABCA4* | pat | Stargardt disease |
| Case of Stargardt Disease Caused by Uniparental Isodisomy John H. Fingert | 10.1001/archopht.124.5.744 | Case report | 1 | *ABCA4* | pat | Stargardt disease |
| Maternal uniparental meroisodisomy in the LAMB3 region of chromosome 1 results in lethal junctional epidermolysis bullosa Y Takizawa | 10.1046/j.1523-1747.1998.00186.x | Case report | 1 | *LAMB3* | mat | Junctional epidermolysis bullosa |
| Complete maternal isodisomy of chromosome 3 in a child with recessive dystrophic epidermolysis bullosa but no other phenotypic abnormalities Hiva Fassihi | 10.1038/sj.jid.5700348 | Case report | 3 | *COL7A1* | mat | Dystrophic epidermolysis bullosa |
| Maternal UPD of chromosome 7 in a patient with Silver-Russell syndrome and Pendred syndrome Chuan Zhang | 10.1002/jcla.23407 | Case report | 7 | *SLC26A4* | mat | Pendred syndrome |
| First maternal uniparental disomy for chromosome 2 with PREPL novel frameshift mutation of congenital myasthenic syndrome 22 in an infant Ping Zhang | 10.1002/mgg3.1144 | Case report | 2 | *PREPL* | mat | Myasthenie |
| Uniparental disomy unveils a novel recessive mutation in POMT2 Brianna N Brun | 10.1016/j.nmd.2018.04.003 | Case report | 14 | *POMT2* | mat | Limb-girdle muscular dystrophies |
| A case of Usher syndrome type IIA caused by a rare USH2A homozygous frameshift variant with maternal uniparental disomy (UPD) in a Chinese family Jiewen Fu | 10.1111/jcmm.15405 | Case report | 1 | *USH2A* | mat | Usher syndrome type IIA |
| Paternal uniparental heterodisomy with partial isodisomy of chromosome 1 in a patient with retinitis pigmentosa without hearing loss and a missense mutation in the Usher syndrome type II gene USH2A Carlo Rivolta | 10.1001/archopht.120.11.1566 | Case report | 1 | *USH2A* | pat | Retinitis pigmentosa |
| Uniparental disomy of chromosome 8 leading to homozygosity of a CYP11B1 mutation in a patient with congenital adrenal hyperplasia: implication for a rare etiology of an autosomal recessive disorder Keiko Matsubara | 10.1507/endocrj.ej13-0509 | Case report | 8 | *CYP11B1* | pat | Congenital adrenal hyperplasia |
| Nijmegen breakage syndrome (NBS) due to maternal isodisomy of chromosome 8 Raymonda Varon | 10.1002/ajmg.a.31540 | Case report | 8 | *NBN* | mat | Nijmegen breakage syndrome |
| Segmental uniparental disomy of chromosome 4 in a patient with methylmalonic acidemia | 10.1002/mgg3.1063 | Case report | 4 | *MMAA* | mat | Methylmalonic acidemia |
| Congenital absence of insulin cells in a neonatal with diabetes mellitus associated with methylmalonic acidemia due to uniparental disomy of chromosome 6 Author links open overlay panelHDorchy | 10.1016/0929-693x(95)90154-u |  | 6 |  |  | Diabetes mellitus associated with methylmalonic acidemia |
| LRBA Deficiency in a Patient With a Novel Homozygous Mutation Due to Chromosome 4 Segmental Uniparental Isodisomy Pere Soler-Palacín | 10.3389/fimmu.2018.02397 | Case report | 4 | *LRBA* | mat | LRBA deficiency |
| Chronic granulomatous disease caused by maternal uniparental isodisomy of chromosome 16 MaríaBravo García-Morato | 10.1016/j.jaip.2017.01.018 | Clinical communication | 16 | *CYBA* | mat | Chronic granulomatous disease |
| New human combined immunodeficiency caused by interferon regulatory factor 4 (IRF4) deficiency inherited by uniparental isodisomy María Bravo García-Morato | 10.1016/j.jaci.2017.12.995 | Case report | 6 | *IRF4* | mat | Primary immunodeficiencies |
| Uniparental isodisomy as a cause of mitochondrial complex I respiratory chain disorder due to a novel splicing NDUFS4 mutation Adrián González-Quintana | 10.1016/j.ymgme.2020.10.008 | Case report | 5 | *NDUFS4* | pat | Mitochondrial complex I respiratory chain disorder |
| CACP syndrome: identification of five novel mutations and of the first case of UPD in the largest European cohort Sara Ciullini Mannurita | 10.1038/ejhg.2013.123 | Review of a case | 1 | *PRG4* | pat | CAPC |
| Uniparental disomy determined by whole-exome sequencing in a spectrum of rare motoneuron diseases and ataxias Dana M Bis | 10.1002/mgg3.285 | Review of a case (96 cases) | 16 | *SPG35* | pat | Spastic paraplegia |
| CD45-deficient severe combined immunodeficiency caused by uniparental disomy Joseph L Roberts | 10.1073/pnas.1202249109 | Case report | 1 | *CD45* | mat | CD45-deficient severe combined immunodeficiency |
| Uniparental Disomy in Cartilage-Hair Hypoplasia Sulisalo T. | PMID: 9156319 | Case report (2 out of 54 uniplex families) | 9 | *CHH* | mat | Cartilage hait hypoplasia |
|  |  |  | 9 | *CHH* |  | Cartilage hait hypoplasia |
| Mandibuloacral Dysplasia Caused by LMNA Mutations and Uniparental Disomy Shaochun Bai | 10.1155/2014/508231 | Case report | 1 | *LMNA* | mat | Mandibuloacral Dysplasia |
| Paternal or Maternal Uniparental Disomy of Chromosome 16 Resulting in Homozygosity of a Mutant Allele Causes Fanconi Anemia Frank X Donovan |  | Case report | 16 | *FANCA *1* | pat | Fanconi anemia |
| Paternal or Maternal Uniparental Disomy of Chromosome 16 Resulting in Homozygosity of a Mutant Allele Causes Fanconi Anemia Frank X Donovan | 10.1002/humu.22962 | Case report | 16 | *FANCP/SLX4 *4* | mat | Fanconi anemia |
| A Unique Mutational Spectrum of MLC1 in Korean Patients With Megalencephalic Leukoencephalopathy With Subcortical Cysts: p.Ala275Asp Founder Mutation and Maternal Uniparental Disomy of Chromosome 22 Sun Ah Choi | 10.3343/alm.2017.37.6.516 | Cohort (5 patients) | 22 | *MLC1* | mat | Megalencephalic leukoencephalopathy with subcortical cysts |
| Achromatopsia: the CNGB3 p.T383fsX mutation results from a founder effect and is responsible for the visual phenotype in the original report of uniparental disomy 14 Wojciech Wiszniewski | 10.1007/s00439-006-0314-y | Cohort (6 patients) | 14 | *CNGB3* | mat | Achromatopsie |
| Coexistence of Mosaic Uniparental Isodisomy and a KCNJ11 Mutation Presenting as Diffuse Congenital Hyperinsulinism and Hemihypertrophy Pınar Kocaay | 10.1159/000446153 |  | 11 | *KCNJ11* | pat | Hyperinsulinaemic hypoglycaemia + BWS |
| Maternal uniparental isodisomy of chromosome 6 reveals a TULP1 mutation as a novel cause of cone dysfunction Susanne Roosing | 10.1016/j.ophtha.2012.12.005 | Cohort (159 patients for CD + 91 patients for CRD) | 6 | *TULP1* | mat | Cone dystrophy |
| Early infantile-onset epileptic encephalopathy 28 due to a homozygous microdeletion involving the WWOX gene in a region of uniparental disomy | 10.1002/humu.23675 |  | 16 | *WWOX* | mat | Epilepsy, Intellectual deficiency, respiratory nnsufficiancy, deafness, skeletal anomalies, CIA |
| Uniparental disomy in cartilage-hair hypoplasia T Sulisalo | PMID: 9156319 | Cohort (54 uniplex families) | 9 |  |  |  |
| Congenital afibrinogenaemia caused by uniparental isodisomy of chromosome 4 containing a novel 15-kb deletion involving fibrinogen Aalpha-chain gene | 10.1038/sj.ejhg.5201207 | Case report | 4 | *FGA* | mat | Afribrinogenemia |
| Two novel homozygous SACS mutations in unrelated patients including the first reported case of paternal UPD as an etiologic cause of ARSACS Laura Anesi | 10.1007/s12031-010-9448-4 | Case report (2 novel mutations, 1/2 = UPD) | 13 | *SACS* | pat | Charlevoix-Saguenay Spastic ataxia |
| Complete paternal isodisomy for chromosome 8 unmasked by lipoprotein lipase deficiency P Benlian | PMID: 8755931 | Case report | 8 | *LPL* | pat | Complete lipoprotein-lipase (LPL) deficiency |
| Paternal uniparental disomy for chromosome 1 revealed by molecular analysis of a patient with pycnodysostosis B D Gelb | 10.1086/301795 | Case report | 1 | *cathepsin* | pat | Pycnodysostosis |
| Segmental uniparental disomy as a rare cause of congenital severe factor XIII deficiency in a girl with only one heterozygous carrier parent Ming-Ching Shen | 10.1080/08880018.2018.1546783 |  | 6 | *F13A1* | mat | Congenital severe factor (F) XIII deficiency |
| Paternal isodisomy for chromosome 2 as the cause of Crigler–Najjar type I syndrome François M Petit | 10.1038/sj.ejhg.5201342 | Case report | 2 | *UGT1A1* | pat | Crigler Najjar |
| Chediak-Higashi syndrome associated with maternal uniparental isodisomy of chromosome 1 R Dufourcq-Lagelouse | 10.1038/sj.ejhg.5200355 | Case report | 1 | *LYST* | mat | Chediak Higashi |
| Isodisomy of chromosome 7 in a patient with cystic fibrosis: could uniparental disomy be common in humans? R Voss | PMID: 2570528 | Case report | 7 | CFTR | mat | Cystic fibrose |
| Hereditary Sensory and Autonomic Neuropathy 2B Caused by a Novel RETREG1 Mutation (c.765dupT) and Paternal Uniparental Isodisomy of Chromosome 5 Geun-Young Park | 10.3389/fgene.2019.01085 | Case report | 5 | *RETREG1* | pat | Hereditary Sensory and Autonomic Neuropathy 2B |
| Unusual Stüve-Wiedemann syndrome with complete maternal chromosome 5 isodisomy Mariarosa A B Melone | 10.1002/acn3.126 |  | 5 | *LIFR* | mat | Stuve-Wiedemann syndrome |
| Complete maternal isodisomy of chromosome 5 in a Japanese patient with Netherton syndrome Sanae Numata | 10.1038/jid.2013.398 | Case report | 5 | *SPINK5* | mat | Netherton syndrome |
| Tyrosinemia type 1 and Angelman syndrome due to paternal uniparental isodisomy 15 Irene Ferrer-Bolufer | 10.1007/s10545-009-9014-9 | Review | 15 | *FAH* | pat | tyrosinemia type 1 + AS |
| Placental mesenchymal dysplasia and fetal renal-hepatic-pancreatic dysplasia: androgenetic-biparental mosaicism and pathogenesis of an autosomal recessive disorder Raj P Kapur | 10.2350/12-12-1281-OA.1 | Case report | 15 | *SNRPN* | pat | Renal-hepatic-pancreatic dysplasia, in a pregnancy complicated by PMD and ABM |
| Blended phenotype of AP4E1 deficiency and Angelman syndrome caused by paternal isodisomy of chromosome 15 Hiroaki Murakami | 10.1016/j.braindev.2019.12.008 | Case report | 15 | *AP4E1* | pat | AP4E1 deficiency and Angelman syndrome |
| Autosomal recessive cystinuria caused by genome-wide paternal uniparental isodisomy in a patient with Beckwith-Wiedemann syndrome Y Ohtsuka | 10.1111/cge.12496 | Case report | 11 | *SLC7A9* | pat | BWS + cystinurie |
| Prader-Willi syndrome and Tay-Sachs disease in association with mixed maternal uniparental isodisomy and heterodisomy 15 in a girl who also had isochromosome Xq Susan Zeesman | 10.1002/ajmg.a.36790 | Case report | 15 + isoXq | *HEXA* | *mat hUPD for PWS + iUPDmat for HEXA* | Tay Sachs disease + PWS |
| Detection of uniparental isodisomy in autosomal recessive mitochondrial DNA depletion syndrome by high-density SNP array analysis Ganka V Douglas | 10.1038/jhg.2011.112 | Case report (2 cases) | 22 | *TYMP* | mat | Mitochondrial DNA (mtDNA) depletion syndrome |
|  |  |  | 2 | *DGUOK* | pat | Mitochondrial DNA (mtDNA) depletion syndrome |
| Recessive congenital methemoglobinemia caused by a rare mechanism: maternal uniparental heterodisomy with segmental isodisomy of a chromosome 22 Yu-Hsiu Huang | 10.1016/j.bcmd.2012.05.005 | Case report | 22 | *CYB5R3* | mat | Recessive congenital methemoglobinemia (RCM) |
| Glucose metabolism and insulin secretion in a patient with ABCC8 mutation and Fanconi-Bickel syndrome caused by maternal isodisomy of chromosome 3 T L Hoffman | 10.1111/j.1399-0004.2007.00802.x | Case report | 3 | *GLUT2* | mat | Fanconi-Bickel syndrome (FBS) |
| Segmental uniparental isodisomy of chromosome 6 causing transient diabetes mellitus and merosin-deficient congenital muscular dystrophy Raissa Coelho Andrade | 10.1002/ajmg.a.36716 | Case report | 6 | *LAMA1* | pat | Merosin-deficient congenital muscular dystrophy type 1A (MDC1A) + TNDM |
| Mosaic segmental uniparental isodisomy and progressive clonal selection: a common mechanism of late onset β-thalassemia major Cornelis L Harteveld | 10.3324/haematol.2012.065219 | Case report | 11 | *IGF2 + H19* | pat | BWS + B thalassemie |
| A girl with infantile neuronal ceroid lipofuscinosis caused by novel PPT1 mutation and paternal uniparental isodisomy of chromosome 1 Yo Niida | 10.1016/j.braindev.2016.01.004 | Case report | 1 | *PPT1* | pat | Neuronal ceroid lipofuscinosis |
| Paternal isodisomy for chromosome 7 is compatible with normal growth and development in a patient with congenital chloride diarrhea P Höglund | PMID: 7942853 | Case report | 7 | CFTR | pat | Congenital chloride diarrhea |
| Isodisomy of chromosome 6 in a newborn with methylmalonic acidemia and agenesis of pancreatic beta cells causing diabetes mellitus M J Abramowicz | 10.1172/JCI117339 | Case report | 6 | *MUT* | pat | Methylmalonic acidemia and agenesis of pancreatic beta cells causing diabetes mellitus |
| Infantile neuroaxonal dystrophy caused by uniparental disomy Joyce Solomons | 10.1111/dmcn.12327 | Case report | 22 | *PLA2G6* | mat | Infantile neuroaxonal dystrophy |
| A case of early-onset epileptic encephalopathy with a homozygous TBC1D24 variant caused by uniparental isodisomy Mitsuko Nakashima | 10.1002/ajmg.a.61056 | Case report | 16 | *TBC1D24* | mat | Epileptic encephalopathy |
| Primary congenital glaucoma caused by the homozygous F261L CYP1B1 mutation and paternal isodisomy of chromosome 2 M-P López-Garrido | 10.1111/j.1399-0004.2009.01242.x | Case report | 2 | *CYP1B1* | pat | Primary congenital glaucoma |
| Maternal uniparental isodisomy is responsible for serious molybdenum cofactor deficiency Hakan Gümüş | 10.1111/j.1469-8749.2010.03724.x | Case report | 6 | *MOCS1* | mat | Molybdenum cofactor deficiency |
| Paternal isodisomy of chromosome 3 unmasked by autosomal recessive microcoria-congenital nephrosis syndrome (Pierson syndrome) in a child with no other phenotypic abnormalities Verena Matejas | 10.1002/ajmg.a.34214 | Case report | 3 | *LAMB2* | pat | Microcoria-congenital nephrosis syndrome (Pierson syndrome) |
| Bloom syndrome and maternal uniparental disomy for chromosome 15 T Woodage | PMID: 7912890 | Case report | 15 | *BLM* | mat | BWS + bloom syndrome |
| Paternal uniparental isodisomy of chromosome 22 in a patient with metachromatic leukodystrophy Yo Niida | 10.1038/jhg.2012.97 | Case report | 22 | *ARSA* | pat | Metachromatic leukodystrophy |
| Paternal isodisomy of chromosome 2 as a cause of long chain 3-hydroxyacyl-CoA dehydrogenase (LCHAD) deficiency Berivan Baskin | 10.1002/ajmg.a.33462 | Case report | 2 | *HADHA* | pat | Long chain 3-hydroxyacyl-CoA dehydrogenase (LCHAD) deficiency |
| Application of multiplex ligation-dependent probe amplification, and identification of a heterozygous Alu-associated deletion and a uniparental disomy of chromosome 1 in two patients with 3-hydroxy-3-methylglutaryl-CoA lyase deficiency Yuka Aoyama | 10.3892/ijmm.2015.2184 | Case report | 1 | *HMGCL* | pat | 3-hydroxy-3-methylglutaryl-CoA lyase deficiency |
| Complete paternal uniparental isodisomy of chromosome 1 resulting in Herlitz junctional epidermolysis bullosa | 10.1111/j.1365-2230.2004.01660.x | Case report | 1 | *LAMB3* | pat | Herlitz junctional epidermolysis bullosa |
| Donnai-Barrow syndrome (DBS/FOAR) in a child with a homozygous LRP2 mutation due to complete chromosome 2 paternal isodisomy Sibel Kantarci | 10.1002/ajmg.a.32381 | Case report | 2 | *LRP2* | pat | Donnai-Barrow syndrome (DBS/FOAR) |
| Uniparental isodisomy 6 associated with deficiency of the fourth component of complement T R Welch | 10.1172/JCI114760 | Case report | 6 | *MCH* | pat | Deficiency of the fourth component of complement |
| Maternal isodisomy of the telomeric end of chromosome 2 is responsible for a case of primary hyperoxaluria type 1 Françoise Chevalier-Porst | 10.1002/ajmg.a.30375 | Case report | 2 | *AGXT* | mat | Primary hyperoxaluria type 1 |
| Paternal isodisomy for chromosome 5 in a child with spinal muscular atrophy L M Brzustowicz | PMID: 8116617 | Case report | 5 | *SMA* | pat | Spinal muscular atrophy |
| Fatal malonyl CoA decarboxylase deficiency due to maternal uniparental isodisomy of the telomeric end of chromosome 16 S Malvagia | 10.1111/j.1469-1809.2007.00373.x | Case report | 16 | *MLYCD* | mat | Fatal malonyl CoA decarboxylase deficiency |
| Trisomic rescue causing reduction to homozygosity for a novel ABCA12 mutation in harlequin ichthyosis D Castiglia | 10.1111/j.1399-0004.2009.01198.x | Case report | 2 | *ABCA12* | pat | Harlequin ichthyosis |
| Third case of paternal isodisomy for chromosome 7 with cystic fibrosis: a new patient presenting with normal growth Cedric Le Caignec | 10.1002/ajmg.a.31999 | Case report (3 cases) | 7 | *CFTR* | pat | Cystic fibrosis |
|  |  |  | 7 | *CFTR* | pat | Cystic fibrosis |
|  |  |  | 7 | *CFTR* | pat | Cystic fibrosis |
| Maternal uniparental disomy of chromosome 2 in a patient with a DGUOK mutation associated with hepatocerebral mitochondrial DNA depletion syndrome Coralie Haudry | 10.1016/j.ymgme.2012.10.008 | Case report | 2 | *DCGUOK* | mat | Hepatocerebral mitochondrial DNA depletion syndrome |
| Mutation report: complete paternal uniparental isodisomy of chromosome 1: a novel mechanism for Herlitz junctional epidermolysis bullosa Y Takizawa | 10.1046/j.1523-1747.2000.00052.x | Case report | 1 | *LAMC2* | pat | Herlitz junctional epidermolysis bullosa |
| Maternal uniparental heterodisomy with partial isodisomy of a chromosome 2 carrying a splice acceptor site mutation (IVS9-2A>T) in ALS2 causes infantile-onset ascending spastic paralysis (IAHSP) Thilo Herzfeld | 10.1007/s10048-008-0148-y | Case report | 2 | *ALS2* | mat | Infantile-onset ascending spastic paralysis (IAHSP) |
| Maternal segmental disomy in Leigh syndrome with cytochrome c oxidase deficiency caused by homozygous SURF1 mutation A K J van Riesen | 10.1055/s-2006-924227 | Case report | 9 | *SURF1* | mat | Leigh syndrome |
| Uniparental disomy of the entire X chromosome in a female with Duchenne muscular dystrophy F Quan | PMID: 8981959 | Case report | X | *DMD* | mat | Duchenne muscular dystrophy |
| Tyrosinemia type 1 and Angelman syndrome due to paternal uniparental isodisomy 15 Irene Ferrer-Bolufer | 10.1007/s10545-009-9014-9 | Review | 15 | *FAH* | pat | Tyrosinemia type 1 + AS |
| Uniparental Disomy of Chromosome 2 Unmasks New ITGA6 Recessive Mutation and Results in a Lethal Junctional Epidermolysis Bullosa in a Newborn Rebecca Higgins | 10.2340/00015555-3313 | Case report | 2 | *ITGA6* | mat | Junctional Epidermolysis Bullosa |
| Uniparental disomy of chromosome 1 unmasks recessive mutations of PPT1 in a boy with neuronal ceroid lipofuscinosis type 1 Lorena Travaglini | 10.1016/j.braindev.2016.08.010 | Case report | 1 | *PPT1* | pat | Neuronal ceroid lipofuscinosis type 1 |
| Anderson's disease/chylomicron retention disease in a Japanese patient with uniparental disomy 7 and a normal SAR1B gene protein coding sequence Tomoo Okada | 10.1186/1750-1172-6-78 | Case report | 7 | *SAR1B* | mat | Anderson's Disease (AD)/Chylomicron Retention Disease (CMRD) |
| Novel mutation of the perforin gene and maternal uniparental disomy 10 in a patient with familial hemophagocytic lymphohistiocytosis Fatma Al-Jasmi | 10.1097/MPH.0b013e31817580fd | Case report | 10 | *perforin* | mat | Familial hemophagocytic lymphohistiocytosis |
| Primary T-cell immunodeficiency with immunodysregulation caused by autosomal recessive LCK deficiency Fabian Hauck | 10.1016/j.jaci.2012.07.029 | Case report | 1 | *LCK* | mat | Primary T-cell immunodeficiency with immunodysregulation |
| A new structural rearrangement associated to Wolfram syndrome in a child with a partial phenotype Francesca M Elli | 10.1016/j.gene.2012.06.077 | Case report | 4 | *WFS1* | pat | Wolfram syndrome |
| Congenital insensitivity to pain with anhidrosis (CIPA): novel mutations of the TRKA (NTRK1) gene, a putative uniparental disomy, and a linkage of the mutant TRKA and PKLR genes in a family with CIPA and pyruvate kinase deficiency Y Indo | 10.1002/humu.1192 | Case report | 1 | *TRKA* | pat | Congenital insensitivity to pain with anhidrosis |
| Uniparental disomy as a cause of spinal muscular atrophy and progressive myoclonic epilepsy: phenotypic homogeneity due to the homozygous c.125C>T mutation in ASAH1 Beatriz G Giráldez | 10.1016/j.nmd.2014.11.007 | Case report | 8 | *ASAH1* | pat | Spinal muscular atrophy and progressive myoclonic epilepsy |
| Uniparental disomy in steroid 5alpha-reductase 2 deficiency B Chávez | 10.1210/jcem.85.9.6786 | Case report | 2 | *E197D* | pat | Steroid 5alpha-reductase 2 deficiency |
| A novel homozygous mutation of GJC2 derived from maternal uniparental disomy in a female patient with Pelizaeus-Merzbacher-like disease Keiko Shimojima | 10.1016/j.jns.2013.04.017 | Case report | 1 | *GJC2* | mat | Pelizaeus-Merzbacher-like disease |
| Maternal uniparental disomy of chromosome 1 with reduction to homozygosity of the LAMB3 locus in a patient with Herlitz junctional epidermolysis bullosa L Pulkkinen | 10.1086/515524 | Case report | 1 | *LAMB3* | mat | Herlitz junctional epidermolysis bullosa |
| Maternal uniparental disomy of chromosome 4 and homozygous novel mutation in the WFS1 gene in a paediatric patient with Wolfram syndrome D T Papadimitriou | 10.1016/j.diabet.2015.06.003 | Case report | 4 | *WFS1* | mat | Wolfram syndrome |
| A uniparental isodisomy event introducing homozygous pathogenic variants drives a multisystem metabolic disorder Eileen G Daniels | 10.1101/mcs.a004457 | Case report | 12 | *PFKM,PUS1* | pat | Tarui syndrome, myopathy, lactic acidosis, and sideroblastic anemia (MLASA) |
| Lysinuric protein intolerance with homozygous SLC7A7 mutation caused by maternal uniparental isodisomy of chromosome 14 Eungu Kang | 10.1038/s10038-019-0657-6 | Case report | 14 | *SLC7A7* | mat | Lysinuric protein intolerance |
| Primary congenital glaucoma due to paternal uniparental isodisomy of chromosome 2 and CYP1B1 deletion Emmanuelle Souzeau | 10.1002/mgg3.774 | Case report | 2 | *CYP1B1* | pat | Primary congenital glaucoma |
| Maternal uniparental isodisomy of chromosome 6 unmasks a novel variant in TULP1 in a patient with early onset retinal dystrophy Emmanuelle Souzeau | PMID: 30090012 | Case report | 6 | *TULP1* | mat | Retinal dystrophy |
| Congenital hyperinsulinism and glucose hypersensitivity in homozygous and heterozygous carriers of Kir6.2 (KCNJ11) mutation V290M mutation: K(ATP) channel inactivation mechanism and clinical management Karen J Loechner | 10.2337/db10-0731 | Case report | 11 | *kcnj11* | pat | Congenital Hyperinsulinism and Glucose Hypersensitivity |
| Maternal chromosome 4 heterodisomy/isodisomy and Bβ chain Trp323X mutation resulting in severe hypodysfibrinogenaemia Qiulan Ding | 10.1160/TH12-02-0088 | Case report | 4 | *FGB* | mat/i | Severe hypodysfibrinogenaemia |
| Uniparental disomy of chromosome 1 causing concurrent Charcot-Marie-Tooth and Gaucher disease Type 3 W S Benko | 10.1212/01.wnl.0000305963.37449.32 | Case report | 1 | *GBA + MPZ* | pat | Charcot-Marie-Tooth and Gaucher disease Type 3 |
| Whole exome sequencing in congenital pain insensitivity identifies a novel causative intronic NTRK1-mutation due to uniparental disomy Ingo Kurth | 10.1002/ajmg.b.32458 | Case report | 1 | *NTRK1* |  | Congenital insensitivity to pain and anhidrosis (CIPA) |
| Uniparental disomy as a mechanism for CERS3-mutated autosomal recessive congenital ichthyosis S Polubothu | 10.1111/bjd.16999 | Case report | 15 | *CERS3* | mat | PWS and CERS3-relatedARC |
| Partial uniparental isodisomy of chromosome 16 unmasks a deleterious biallelic mutation in IFT140 that causes Mainzer-Saldino syndrome Benjamin M Helm | 10.1186/s40246-017-0111-9 | Case report | 16 | *IFT140* | mat | Mainzer-Saldino syndrome |
| Paternal uniparental isodisomy of chromosome 6 causing a complex syndrome including complete IFN-gamma receptor 1 deficiency Carolina Prando | 10.1002/ajmg.a.33291 | Review | 6 | *IFNGR1* | pat | IFN-gamma receptor 1 deficiency |
| Maternal isodisomy for chromosome 9 causing homozygosity for a novel FOXE1 mutation in syndromic congenital hypothyroidism Mireille Castanet | 10.1210/jc.2010-0275 | Case report | 9 | *FOXE1* | mat | Congenital hypothyroidism |
| Homozygous leptin receptor mutation due to uniparental disomy of chromosome 1: response to bariatric surgery Johanne Le Beyec | 10.1210/jc.2012-2779 | Case report | 1 | *LEPR* | pat | In this case, gastroplasty may be partially effective for weight control as illustrated. |
| An unusual presentation of macular corneal dystrophy associated with uniparental isodisomy and a novel Leu173Pro mutation Vivek S Yellore | 10.1080/13816810701407925 | Case report | 16 | *CHST6* | mat | Macular corneal dystrophy |
| Complement factor H deficiency and endocapillary glomerulonephritis due to paternal isodisomy and a novel factor H mutation L Schejbel | 10.1038/gene.2010.63 | Case report | 1 | *CFH* | pat | Complement factor H deficiency and endocapillary glomerulonephritis |
| Two homozygous mutations in the exon 5 of BCKDHB gene that may cause the classic form of maple syrup urine disease Ling Su | 10.1007/s11011-017-9959-6 | Case report | 6 | *BCKDHB* | mat | Maple syrup urine disease (MSUD) |
| Atypical giant axonal neuropathy arising from a homozygous mutation by uniparental isodisomy S Miyatake | 10.1111/cge.12455 | Case report | 16 | *GAN* | mat | Atypical giant axonal neuropathy |
| Genotype/phenotype correlations in complement factor H deficiency arising from uniparental isodisomy Valerie Wilson | 10.1053/j.ajkd.2013.05.020 | Case report | 1 | *CFH* | pat | Complement factor H deficiency |
| Paternal isodisomy of chromosome 5 in a patient with recessive multiple epiphyseal dysplasia Mónica Martínez García | 10.1002/ajmg.a.36378 | Case report | 5 | *SLC26A2* | pat | Epiphyseal dysplasia |
| Uniparental Isodisomy of Chromosome 1 Unmasking an Autosomal Recessive 3-Beta Hydroxysteroid Dehydrogenase Type II-Related Congenital Adrenal Hyperplasia Karin Panzer | 10.4274/jcrpe.3680 | Case report | 1 | *HSD3B2* | NA | 3-Beta Hydroxysteroid Dehydrogenase Type II-Related Congenital Adrenal Hyperplasia |
| Chediak-Higashi syndrome with early developmental delay resulting from paternal heterodisomy of chromosome 1 | 10.1002/ajmg.a.33389 | Case report | 1 | *CHS1F* | pat | Chediak-Higashi syndrome |
| Uniparental disomy for chromosome 6 results in steroid 21-hydroxylase deficiency: evidence of different genetic mechanisms involved in the production of the disease A U López-Gutiérrez | 10.1136/jmg.35.12.1014 |  | 6 | *CYP21* | pat | Congenital adrenal hyperplasia (CAH) |
| Intrauterine growth retardation associated with maternal uniparental disomy for chromosome 6 unmasked by congenital adrenal hyperplasia R P Spiro | 10.1203/00006450-199911000-00004 | Case report | 6 | *CYP21* | mat | Congenital adrenal hyperplasia (CAH) |
| Severe phenotype of severe combined immunodeficiency caused by adenosine deaminase deficiency in a patient with a homozygous mutation due to uniparental disomy Joyce Geelen | 10.1016/j.jaci.2012.11.006 | Case report | 20 | *ADA* | pat | Denosinedeaminase deficiency (ADA-SCID) |
| Neonatal onset autosomal dominant polycystic kidney disease (ADPKD) in a patient homozygous for a PKD2 missense mutation due to uniparental disomy M Losekoot | 10.1136/jmedgenet-2011-100452 | Case report | 4 | *PKD2* | mat | Polycystic kidney disease |
| {beta}-thalassemia major evolution from {beta}-thalassemia minor is associated with paternal uniparental isodisomy of chromosome 11p15 Jan-Gowth Chang | 10.3324/haematol.12195 | Case report | 11 | *HBB* | pat | Major beta-thalassemia |
| Uniparental disomy of chromosome 13q causing homozygosity for the 35delG mutation in the gene encoding connexin26 (GJB2) results in prelingual hearing impairment in two unrelated Spanish patients A Alvarez | 10.1136/jmg.40.8.636 | Case report (2 cases) | 13 | *GJB2* | mat | Prelingual hearing impairment |
|  |  |  | 13 | *GJB2* | mat | Prelingual hearing impairment |
| Complete maternal isodisomy of chromosome 3 in a child with recessive dystrophic epidermolysis bullosa but no other phenotypic abnormalities Hiva Fassihi | 10.1038/sj.jid.5700348 | Case report | 3 | *COL7A1* | mat | Dystrophic epidermolysis bullosa |
| Complete maternal isodisomy causing reduction to homozygosity for a novel LAMB3 mutation in Herlitz junctional epidermolysis bullosa Marco Castori | 10.1016/j.jdermsci.2008.02.006 | Case report | 1 | *LAMB3* | mat | Herlitz junctional epidermolysis bullosa |

**Table S5: Distribution of the UPD cases in the literature per chromosome. The OMIM list of morbid genes with a recessive inheritance is given (11/2021).**

| **Chromosome** | **#UPD cases** | **#Recessive OMIM genes** |
| --- | --- | --- |
| 1 | 35 | 241 |
| 2 | 22 | 181 |
| 3 | 6 | 150 |
| 4 | 9 | 100 |
| 5 | 7 | 100 |
| 6 | 14 | 118 |
| 7 | 8 | 96 |
| 8 | 7 | 88 |
| 9 | 6 | 101 |
| 10 | 1 | 95 |
| 11 | 8 | 173 |
| 12 | 4 | 118 |
| 13 | 4 | 44 |
| 14 | 5 | 71 |
| 15 | 9 | 87 |
| 16 | 18 | 111 |
| 17 | 4 | 143 |
| 18 | 0 | 38 |
| 19 | 0 | 123 |
| 20 | 1 | 55 |
| 21 | 0 | 32 |
| 22 | 5 | 51 |
| X | 1 | 2 |
| **TOTAL** | **174** | **2318** |
