# Supplementary material for: Unexpected Inheritance Patterns in a Large Cohort of Patients with a Suspected Ciliopathy
Source: Hum Mutat. 2023 Aug 9;2023:2564200. doi: 10.1155/2023/2564200 (PMC11918889; doi:10.1155/2023/2564200)
Supplement: Supplementary Materials — Supplementary data includes the following figures and tables. Figure S1: flowchart illustrating the distribution of positive cases among the cohort. Figure S2: pie chart illustrating the distribution of the 499 BBS-positive cases and their genes in the cohort. Figure S3: microsatellite analysis for 3 families, including 2 UPD (A and B) and 1 de novo (C). Table S1: analyses performed on individuals carrying an UPD or a de novo variant. Table S2: list of variations identified and their ACMG classification. Table S3: list of pathologies and related genes identified in positive cases in the category named “other” together with detailed clinical data. Table S4: literature review raw data including article title, DOI/PMID, gene and chromosome, parental origin, and the disease. Table S5: distribution of the UPD cases in the literature per chromosome. [file 2564200.f1.zip › SupplementaryTable3_OtherDiseases.pdf]

Family ID	Age at last examination	Gender	Family position	Consanguinity	Gene	Pathology	Publication of our laboratory	Prenatal features	Retinitis pigmentosa HP:0000510	Nystagmus HP:0000639	Photophobia HP:0000613	Other ophthalmic features	Obesity HP:0001513	Polydactyly HP:0010442	Brachydactyly HP:0200055	Syndactyly HP:0001159	Intellectual disability (severity) HP:0001249	Learning difficulties HP:0001328	Speech delay HP:0000750	Slow ideation	Motor delay HP:0001270	Renal anomalies HP:0000077	Renal insufficiency HP:0000083	Hyperechogenic kidneys HP:0004719	Multicystic dysplasia HP:0004719	Other renal features	Hypogonadism HP:0000135	Deafness HP:0000365	Cardiomyopathy HP:0001638	Congenital heart anomaly	Diabetes HP:0000819	Other
XII.7	16 y	M	sibling	no	VPS15/PIK3R4	Ciliopathy	Stoetzel (2016) 10.1038/ncomms13586	NA	yes	no	yes	NA	no	no	yes	no	no	NA	yes	no	yes	yes	yes	NA	NA	NA	NA	NA	NA	NA	NA	growth retardation, dysmorphism
XII.7	33 y	M	proband	no	VPS15/PIK3R4	Ciliopathy	Stoetzel (2016) 10.1038/ncomms13586	NA	yes	NA	NA	cataract	yes	yes	yes	NA	NA	yes	NA	NA	NA	yes	yes	no	yes	NA	NA	NA	yes (dilated)	NA	no	growth retardation, dysmorphism
XII.7	27 y	F	sibling	no	VPS15/PIK3R4	Ciliopathy	Stoetzel (2016) 10.1038/ncomms13586	no	yes	no	yes	NA	no	no	yes	no	no	no	NA	NA	NA	yes	yes	NA	yes	NA	yes	no	NA	NA	no	dental anomalies, enuresia, dysmorphism
XVI.14	37 y	M	proband	yes	TMEM231	Joubert syndrome / Meckel syndrome		NA	yes	yes	no	cataract	yes	yes	NA	yes	yes	NA	yes	NA	yes	yes	yes	NA	NA	NA	NA	NA	NA	NA	no	macrocephaly, angioma
XV.12	23 y	F	proband	no	FAM161A	Retinitis pigmentosa		no	yes	no	NA	NA	yes	no	no	yes	no	yes	yes	yes	yes	no	no	no	no	NA	no	NA	no	no	no	hyperinsulinemia
XIV.28	6 y	M	proband	no	FMR1	Fragile X syndrome		no	NA	yes	NA	NA	yes	no	yes	no	yes	NA	yes	NA	yes	no	no	no	no	NA	yes	NA	NA	NA	NA	NA
CXXVII.20_EADN	NA	M	proband	no	WDR19	Nephronophthisis		NA	yes	NA	yes	NA	NA	NA	NA	NA	NA	NA	NA	NA	NA	yes	yes	NA	NA	acute renal insufficiency at 4 years old	NA	NA	NA	NA	NA	NA
XIV.10-23	9 y	M	proband	yes	TRAF3IP1/IFT54	Senior-Loken syndrome		NA	yes	NA	NA	NA	yes	yes	NA	NA	yes	NA	NA	NA	NA	yes	no	yes	NA	Abnormal tubulointerstitial morphology	NA	NA	no	yes	NA	macrocephaly
XIV.10-23	12 y	F	sibling	yes	TRAF3IP1/IFT54	Senior-Loken syndrome		NA	yes	yes	NA	NA	yes	yes	NA	NA	yes	NA	NA	NA	NA	yes	yes	NA	NA	unilateral renal agenesis	NA	yes	NA	yes	NA	macrocephaly
XIV.10-23	18 y	F	sibling	yes	TRAF3IP1/IFT54	Senior-Loken syndrome		NA	yes	NA	NA	NA	NA	no	NA	NA	no	NA	NA	NA	NA	yes	yes	NA	NA	Abnormal tubulointerstitial morphology	NA	yes	NA	NA	NA	macrocephaly
XVIII.28	10 y	F	proband	yes	SMC1A	Cornelia de Lange syndrome		NA	NA	NA	NA	ptosis	yes	no	yes	no	yes (moderate)	NA	NA	NA	NA	NA	NA	NA	NA	NA	NA	NA	NA	NA	NA	NA
XII.4	fetus	M	proband	no	GLI3	Pallister-Hall syndrome		yes	NA	NA	NA	NA	yes	yes	NA	yes	NA	NA	NA	NA	NA	no	NA	no	no	nephromegaly, adrenal glands hypertrophy	no	NA	no	no	NA	corpus callosum agenesis, pilonidal abscess, left stomach, median liver, polysplenia, annular pancreas, abnormal
XV.30	44 y	F	proband	NA	IFT140	Retinitis pigmentosa	Geoffroy (2018) 10.1002/humu.23539	NA	yes	NA	NA	no	NA	no	yes	NA	NA	NA	NA	NA	NA	yes	yes	NA	NA	Abnormal tubulointerstitial morphology	NA	NA	NA	NA	NA	NA
XXIII.7	12 y	F	proband	no	CNGA3	Achromatopsia		no	yes	no	yes	NA	yes	no	no	no	yes (mild)	yes	no	yes	no	no	no	no	no	NA	no	NA	no	no	no	NA
XVIII.30	fetus	M	proband	yes	CC2D2A	Meckel syndrome		yes (unilateral renal cysts)	NA	NA	NA	NA	NA	NA	NA	NA	NA	NA	NA	NA	NA	yes	NA	NA	yes	NA	NA	NA	NA	yes	NA	Dandy-Walker malformation
IX.27	5 y	M	proband	yes	IQCE	Polydactyly	Estrada-Cuzcano (2019) 10.1002/humu.23924	NA	yes	NA	NA	NA	yes	yes	yes	yes	NA	NA	NA	NA	NA	NA	NA	NA	NA	NA	NA	NA	NA	NA	NA	NA
XX.20	14 days of life	M	proband	no	IQCE	Polydactyly	Estrada-Cuzcano (2019) 10.1002/humu.23924	no	NA	NA	NA	NA	yes	yes	NA	yes	NA	NA	NA	NA	NA	no	NA	NA	NA	NA	NA	no	no	no	NA	NA
XX.20	1.5 y	F	sibling	no	IQCE	Polydactyly	Estrada-Cuzcano (2019) 10.1002/humu.23924	no	NA	NA	NA	NA	NA	yes	NA	NA	NA	NA	NA	NA	NA	NA	NA	NA	NA	NA	NA	NA	NA	NA	NA	NA
XX.20	14 days of life	F	sibling	no	IQCE	Polydactyly	Estrada-Cuzcano (2019) 10.1002/humu.23924	no	NA	NA	NA	NA	no	yes	NA	NA	NA	NA	NA	NA	NA	no	NA	NA	NA	NA	NA	no	no	no	NA	NA
XIII.20	NA	M	proband	NA	PNPLA6	Oliver–McFarlane syndrome	Hufnagel (2015) 10.1136/jmedgenet-2014-102856	NA	NA	NA	NA	NA	NA	NA	NA	NA	NA	NA	NA	NA	NA	NA	NA	NA	NA	NA	NA	NA	NA	NA	NA	NA
XIX.22	7 y	M	proband	no	LAMA1	Poretti-Boltshauser syndrome		no	no	NA	NA	myopia, strabismus	no	no	NA	NA	no	no	yes	NA	yes	no	no	no	no	NA	NA	NA	no	no	NA	molar tooth, cerebellum atrophy, agenesis of the vermis, oculomotor apraxia
XX.25	22 y	M	proband	yes	TRAF3IP1/IFT54	Senior-Loken syndrome		no	yes	NA	NA	hemeralopia	NA	NA	NA	NA	yes	NA	NA	NA	NA	yes	yes	NA	NA	NA	NA	NA	NA	NA	NA	NA
XX.25	NA	M	sibling	yes	TRAF3IP1/IFT54	Senior-Loken syndrome		NA	NA	NA	NA	NA	NA	yes	NA	NA	NA	NA	NA	NA	NA	NA	NA	NA	NA	NA	NA	NA	NA	NA	NA	NA
XX.25	10 y	M	sibling	yes	TRAF3IP1/IFT54	Senior-Loken syndrome		yes (polydactyly)	yes	NA	NA	NA	yes	yes	NA	NA	no	no	NA	NA	NA	yes	yes	NA	NA	NA	NA	NA	no	no	NA	NA
XXIII.28	6 y	F	proband	yes	VPS13B	Cohen syndrome		no	yes	no	NA	NA	yes	no	yes	yes	yes (moderate)	yes	yes	yes	NA	NA	NA	NA	NA	NA	NA	no	no	NA	no	NA
XVI.31	18 y	F	proband	no	TMEM231	Joubert syndrome / Meckel syndrome		yes (hyperechogenic kidneys)	yes	no	NA	strabismus	no	no	NA	NA	yes (moderate)	yes	yes	yes	yes	yes	yes	yes	no	Reduced renal corticomedullary differentiation	no	no	no	no	no	partial vermis agenesis
XXV.3	28 y	F	proband	no	WDR19	Senior-Loken syndrome		no	yes	no	yes	NA	no	no	NA	NA	no	no	NA	NA	NA	yes	yes	no	no	NA	NA	NA	NA	NA	NA	scoliosis
XXV.3	21 y	F	sibling	no	WDR19	Senior-Loken syndrome		no	yes	no	yes	NA	no	NA	NA	NA	no	yes	NA	NA	NA	no	no	NA	NA	NA	NA	NA	NA	NA	NA	NA
XXII.5	fetus	M	proband	no	HNF1B	Renal cysts and diabetes syndrome		yes	NA	NA	NA	NA	NA	no	NA	NA	NA	NA	NA	NA	NA	yes	NA	yes	yes	NA	NA	NA	no	no	NA	NA
XXIV.31	18 y	M	proband	no	MYO7A	Usher syndrome		no	yes	NA	yes	NA	no	no	no	no	yes (mild)	yes	yes	NA	yes	no	NA	NA	NA	NA	NA	yes	NA	NA	NA	epilepsia
XV.27	17 y	M	sibling	no	IFT140	Retinitis pigmentosa	Geoffroy (2018) 10.1002/humu.23539	no	yes	no	NA	NA	yes	NA	yes	NA	no	no	no	no	no	yes	yes	NA	NA	NA	no	NA	no	NA	no	macrocephaly
XV.27	12 y	M	proband	no	IFT140	Retinitis pigmentosa	Geoffroy (2018) 10.1002/humu.23539	no	yes	no	yes	no	no	no	yes	no	no	no	no	no	no	no	NA	NA	NA	NA	no	NA	no	NA	no	NA
XXV.17	4.5 y	M	proband	no	NPHP1	Senior-Loken syndrome		no	yes	yes	yes	NA	yes	no	no	no	no	no	no	no	no	yes	no	NA	yes	NA	no	NA	no	no	no	NA
XXVI.13	fetus	NA	sibling	yes	TMEM67	Nephronophthisis		yes (bilateral multicystic nephromegaly, Dandy Walker malformation)	NA	NA	NA	NA	NA	NA	NA	NA	NA	NA	NA	NA	NA	yes	NA	yes	yes	NA	NA	NA	NA	NA	NA	hepatomegaly, Dandy Walker malformation
XXVII.14	1 day of life	M	proband	yes	AGT	Renal tubular dysgenesis		yes (hyperechogenic kidneys, anamnios)	NA	NA	NA	NA	NA	no	NA	NA	NA	NA	NA	NA	NA	yes	yes	yes	NA	NA	NA	NA	no	no	NA	NA
XXVII.16	63 y	M	proband	no	IQCB1	Senior-Loken syndrome		NA	yes	NA	NA	NA	NA	NA	NA	NA	NA	NA	NA	NA	NA	yes	yes	NA	yes	NA	NA	NA	NA	NA	NA	NA
XXVII.18	fetus	NA	proband	no	B3GLCT	Peters-plus syndrome		yes (hyperechogenic kidneys and nephromegaly)	NA	NA	NA	NA	NA	no	NA	NA	NA	NA	NA	NA	NA	yes	NA	yes	NA	NA	NA	NA	no	no	NA	corneal opacity, chorio-retinal coloboma
XXVII.21	fetus	F	proband	NA	TMEM67	Meckel syndrome		yes (bilateral multicystic nephromegaly, occipital meningoencephalocele, polysplenia)	NA	NA	NA	NA	no	no	no	no	NA	NA	NA	NA	NA	yes	NA	yes	yes	NA	NA	NA	no	no	NA	occipital meningoencephalocele, common mesentery
XXVI.13	fetus	NA	proband	yes	TMEM67	Meckel syndrome		yes (bilateral multicystic nephromegaly, Dandy Walker malformation)	NA	NA	NA	NA	NA	no	NA	NA	NA	NA	NA	NA	NA	yes	NA	yes	yes	NA	NA	NA	no	no	NA	hepatomegaly, Dandy Walker malformation
XXVIII.7	fetus	NA	proband	no	HNF1B	Renal cysts and diabetes syndrome		yes (bilateral nephromegaly)	NA	NA	NA	NA	NA	no	NA	NA	NA	NA	NA	NA	NA	yes	NA	yes	NA	NA	NA	NA	no	no	NA	NA
XXVIII.20	fetus	NA	proband	no	PKHD1	Polycystic kidney disease		yes (bilateral nephromegaly)	NA	NA	NA	NA	NA	no	no	no	NA	NA	NA	NA	NA	yes	yes	yes	no	NA	NA	NA	no	no	NA	NA
II.21_ALMS	17 y	F	proband	no	YARS1	Infantile-onset multisystem neurologic, endocrine, and pancreatic disease		no	yes	no	no	hemeralopia	no	no	no	no	yes (mild)	yes	yes	no	yes	no	NA	NA	no	NA	yes	no	no	no	NA	NA
XXVIII.29	fetus	NA	proband	no	PKHD1	Polycystic kidney disease		yes (hyperechogenic kidneys)	NA	NA	NA	NA	NA	no	NA	NA	NA	NA	NA	NA	NA	yes	NA	yes	NA	NA	NA	NA	no	NA	NA	NA
XXIX.1	6.5 y	M	proband	no	CPLANE1	Joubert syndrome		NA	NA	NA	NA	NA	NA	NA	NA	NA	yes (severe)	NA	NA	NA	NA	NA	NA	NA	NA	NA	NA	NA	NA	NA	NA	epilepsia, molar tooth
XXIX.31	fetus	NA	proband	no	deletion of HNF1B	Renal cysts and diabetes syndrome		yes (hyperechogenic kidneys)	NA	NA	NA	NA	NA	NA	NA	NA	NA	NA	NA	NA	NA	yes	NA	yes	NA	NA	NA	NA	NA	NA	NA	NA
XXX.6	fetus	M	proband	NA	TMEM70	Mitochondrial complex V (ATP synthase) deficiency, nuclear type 2		yes (intra-uterine growth retardation, dysmorphism, blepharophimosis, cerebellum hypoplasia, cardiomegaly, common mesentery, hypospadias, syndactyly, right ear dysplasia, ileal dilatation, oligoamnios)	NA	NA	NA	NA	NA	no	NA	NA	NA	NA	NA	NA	NA	yes	NA	NA	NA	NA	no	NA	yes	NA	NA	NA
XXX.6	fetus	F	sibling	NA	TMEM70	Mitochondrial complex V (ATP synthase) deficiency, nuclear type 2		yes (intra-uterine growth retardation, dysmorphism, blepharophimosis, cerebellum hypoplasia, cardiomegaly, common mesentery, hypospadias, syndactyly, right ear dysplasia, ileal dilatation, oligoamnios)	NA	NA	NA	NA	NA	no	NA	NA	NA	NA	NA	NA	NA	yes	NA	NA	NA	NA	no	NA	yes	NA	NA	NA
XXX.11	fetus	M	proband	no	GLI3	Polydactyly		yes (postaxial polydactyly)	NA	NA	NA	NA	NA	yes	NA	NA	NA	NA	NA	NA	NA	NA	NA	NA	NA	NA	NA	NA	NA	NA	NA	NA
XXXI.13	5 y	F	proband	no	CNNM4	Jalili syndrome		NA	yes	yes	NA	NA	NA	NA	NA	NA	no	NA	no	NA	yes	NA	NA	NA	NA	NA	NA	yes	NA	NA	NA	tooth enamel dysplasia
XXXI.19	17 y	M	proband	no	AHI1	Joubert syndrome		no	yes	NA	NA	NA	yes	no	no	no	yes (mild)	yes	yes	yes	yes	yes	yes	NA	no	NA	no	no	no	NA	no	NA
XXXII.20	fetus	NA	proband	no	USP9X	Intellectual developmental disorder		yes (postaxial polydactyly, cystic hygroma, cerebral dilatation, Blake pouch cyst, renal pelvic dilatation)	NA	NA	NA	NA	NA	yes	NA	NA	NA	NA	NA	NA	NA	no	NA	no	no	pyelic dilatation	NA	NA	no	no	NA	NA
XXXII.21	6 y	F	proband	no	KIAA0586	Joubert syndrome		NA	NA	NA	NA	NA	no	no	NA	NA	yes	NA	yes	NA	yes	no	no	no	no	acute urinary retention	no	no	no	NA	no	cerebellar dysplasia, bulb atrophy
XXXII.22	fetus	NA	proband	no	deletion of HNF1B	Renal cysts and diabetes syndrome		yes (hyperechogenic kidneys and nephromegaly)	NA	NA	NA	NA	NA	no	NA	NA	NA	NA	NA	NA	NA	yes	NA	yes	yes	NA	NA	NA	no	no	NA	NA
XXXIII.9 BBS	14 y	M	proband	no	RDH12	Leber congenital amaurosis		no	yes	NA	NA	NA	yes	no	no	no	no	no	no	no	no	no	no	NA	NA	NA	no	no	NA	NA	no	NA
XXXIII.25	23 y	M	proband	no	PDE6A	Retinitis pigmentosa		NA	yes	no	NA	no	yes	NA	yes	NA	no	no	no	no	no	yes	no	NA	NA	NA	no	no	no	no	NA	dyspnea, intracranial hypertension
I.31 BBS	21 y	M	proband	NA	IFT140	Retinitis pigmentosa	Geoffroy (2018) 10.1002/humu.23539	NA	yes	NA	NA	NA	no	NA	NA	NA	no	NA	NA	NA	NA	no	NA	NA	NA	NA	NA	NA	NA	NA	NA	NA
XXX.24	6 y	M	proband	yes	RPGRIP1	Leber congenital amaurosis		NA	yes	yes	NA	Leber congenital amaurosis	NA	NA	NA	NA	NA	NA	NA	NA	NA	NA	NA	NA	NA	NA	NA	NA	NA	NA	NA	NA
XXXII.17	29 y	M	proband	no	RP1L1	Retinitis pigmentosa		NA	yes	no	yes	NA	NA	NA	NA	NA	no	no	no	no	no	NA	NA	NA	NA	NA	NA	NA	NA	NA	NA	NA
XXXII.18	fetus	NA	proband	yes	TCTN1	Joubert syndrome		yes (bilateral multicystic nephromegaly, encephalocoele, skeletal anomalies, polydactyly)	NA	NA	NA	NA	NA	yes	NA	NA	NA	NA	NA	NA	NA	yes	NA	yes	yes	NA	NA	NA	no	no	NA	occipital encephalocele, skeletal anomalies
XXVIII.12	21 y	M	proband	NA	ABCA4	Stargardt disease		NA	yes	NA	yes	hemeralopia	no	no	NA	NA	no	yes	yes	NA	no	no		NA	NA	NA	NA	NA	NA	NA	NA	NA
